# Elevated peripheral expression of neuregulin-1 (*NRG1*) mRNA isoforms in clozapine-treated schizophrenia patients

**DOI:** 10.1038/s41398-017-0041-2

**Published:** 2017-12-11

**Authors:** Md Shaki Mostaid, Ting Ting Lee, Gursharan Chana, Suresh Sundram, Cynthia Shannon Weickert, Christos Pantelis, Ian Everall, Chad Bousman

**Affiliations:** 1 Melbourne Neuropsychiatry Center, Department of Psychiatry, The University of Melbourne & Melbourne Health, Parkville, VIC Australia; 2 The Cooperative Research Center (CRC) for Mental Health, Carlton, VIC Australia; 3 Center for Neural Engineering, Department of Electrical and Electronic Engineering, The University of Melbourne, Carlton, VIC Australia; 4 Florey Institute of Neuroscience and Mental Health, The University of Melbourne, Parkville, VIC Australia; 5 Department of Medicine, Royal Melbourne Hospital, Parkville, VIC Australia; 6 NorthWestern Mental Health, Melbourne, VIC Australia; 7 Department of Psychiatry, School of Clinical Sciences, Monash University and Monash Health, Clayton, VIC Australia; 8 Schizophrenia Research Institute, Sydney, NSW Australia; 9 Schizophrenia Research Laboratory, Neuroscience Research Australia, Baker Street, Sydney, NSW Australia; 10 School of Psychiatry, Faculty of Medicine, University of New South Wales, Sydney, NSW Australia; 11 Departments of Medical Genetics, Psychiatry, and Physiology & Pharmacology, University of Calgary, Calgary, AB Canada

## Abstract

Differential expression of neuregulin-1 (*NRG1*) mRNA isoforms and proteins has been reported in schizophrenia, primarily in post-mortem brain tissue. In this study, we examined 12 *NRG1* SNPs, eight *NRG1* mRNA isoforms (type I, type I_(Ig2)_, type II, type III, type IV, EGFα, EGFβ, pan-NRG1) in whole blood, and NRG1-β1 protein in serum of clozapine-treated schizophrenia patients (*N* = 71) and healthy controls (*N* = 57). In addition, using cultured peripheral blood mononuclear cells (PBMC) from 15 healthy individuals, we examined the effect of clozapine on *NRG1* mRNA isoform and protein expression. We found elevated levels of *NRG1* mRNA, specifically the EGFα (*P* = 0.0175), EGFβ (*P* = 0.002) and type I_(Ig2)_ (*P* = 0.023) containing transcripts, but lower NRG1-β1 serum protein levels (*P* = 0.019) in schizophrenia patients compared to healthy controls. However, adjusting for smoking status attenuated the difference in NRG1-β1 serum levels (*P* = 0.050). Examination of clinical factors showed *NRG1* EGFα (*P* = 0.02) and EGFβ (*P* = 0.02) isoform expression was negatively correlated with age of onset. However, we found limited evidence that *NRG1* mRNA isoform or protein expression was associated with current chlorpromazine equivalent dose or clozapine plasma levels, the latter corroborated by our PBMC clozapine exposure experiment. Our SNP analysis found no robust expression quantitative trait loci. Our results represent the first comprehensive investigation of *NRG1* isoforms and protein expression in the blood of clozapine-treated schizophrenia patients and suggest levels of some *NRG1* transcripts are upregulated in those with schizophrenia.

## Introduction

Neuregulin-1 (*NRG1*) is vital for neurodevelopment and plasticity^1^, making it an appealing gene to examine in schizophrenia. This appeal has been weakened by genome-wide association study results that have failed to identify it as a top schizophrenia ‘‘risk’’ gene^2^; questioning the relevance of *NRG1* in schizophrenia^3^. However, the relevance of any gene to schizophrenia should not be determined exclusively on whether sequence variations within the gene meet genome-wide significance but rather on the compendium of knowledge available for that gene. A recent meta-analysis^4^ and systematic review^3^ have showed a number of *NRG1* genetic variants as well as mRNA and protein levels associated with schizophrenia in specific populations or in certain contexts, which could, in part, be attributed to the complex and highly interactive nature of the NRG-ErbB signaling pathway^1,5^. Nevertheless, the bulk of the evidence to date suggests *NRG1* remains an important target for schizophrenia research.

Post-mortem human brain studies in schizophrenia have shown differential expression of *NRG1* mRNA and protein in various brain regions, most notably in dorsolateral prefrontal cortex and hippocampus^6–9^, although other studies of both regions have been negative^10–14^. Similar evidence of differential gene and protein expression in the peripheral tissue of schizophrenia patients is also available. *NRG1* mRNA expression, specifically type II β3 and *NRG1* type III isoforms, have also been shown to be increased in peripheral leukocytes in Portuguese schizophrenia patients^15^ and pan-*NRG1* was shown to be decreased in Chinese schizophrenia patients compared to healthy controls^16,17^. Furthermore, the only two protein studies of *NRG1* in peripheral samples found decreased plasma *NRG1*-β1^18^ and serum Ig-*NRG1* levels^19^ in people with schizophrenia relative to healthy controls. Collectively, these studies suggest *NRG1* may be dysregulated in brain and blood at both the mRNA and protein level in schizophrenia and flag peripheral blood as a potential surrogate for brain *NRG1* dysregulation^20^. However, the number of peripheral blood studies is limited and the influence specific clinical subgroups (e.g., treatment-resistant), genetic variation, medication, lifestyle (e.g., smoking), and/or symptom severity may have on *NRG1* mRNA and protein expression is not clear.

The aim of this study was to address these gaps in the current literature by investigating peripheral mRNA and protein levels of *NRG1* in schizophrenia, as peripheral measures have the potential to serve as biomarkers in the clinical setting. We particularly focused on patients being treated with clozapine. Clozapine is the drug of choice for a subgroup of schizophrenia patients who do not respond to other antipsychotics, referred to as treatment-resistant schizophrenia^21^. Herein, we examined in whole blood, several *NRG1* mRNA isoforms, and *NRG1*-β1 protein levels in serum within those with schizophrenia compared to healthy controls. We also explore how these expression levels relate to symptom severity, age of onset, duration of illness, and *NRG1* genetic variation as well as examine clozapine’s effect on *NRG1* mRNA and protein expression in peripheral blood mononuclear cells (PBMCs) from healthy control subjects.

## Materials and methods

### Participants

#### Clinical samples

Seventy-one individuals with schizophrenia were recruited from inpatient and outpatient clinics located in Melbourne, Australia. Inclusion criteria included: (1) diagnosis of schizophrenia, (2) currently prescribed and taking clozapine, and (3) aged between 18–65 years. Fifty-seven unrelated healthy controls matched for age and sex with similar socio-economic backgrounds were recruited from the general community. Controls with a first-degree family history of psychiatric illness, neurological disease, head injury, seizures, prior or current use of antipsychotic medication, impaired thyroid function and/or substance abuse/dependence were excluded from the study. Participant characteristics are shown in Table 1.

**Table 1:** Demographic data and clinical characteristics of participants

Characteristic	Schizophrenia (*n* = 71)	Controls (*n* = 57)	*P*-value
Age, mean (sd) years	40 (10)	40 (11)	0.702^a^
Gender, *n* (%) males	53 (75)	35 (61)	0.108^b^
RNA integrity number, mean (sd)	8.4 (0.9)	8.7 (0.3)	0.006^a^*
Ancestry, *n* (%) CEU	62 (90)	50 (88)	0.742^b^
Substance use in past three months, *n* (%)			
Tobacco (smoked)	33 (47)	12 (21)	0.003^b^*
Alcohol	59 (83)	55 (97)	0.016^b^*
Cannabis	11 (15)	7 (12)	0.385^b^
Amphetamine	4 (6)	2 (4)	0.439^b^
Cocaine	0 (0)	2 (4)	0.137^b^
Opiates	1 (1)	1 (2)	0.990^b^
Clozapine plasma level, mean (sd) µg/L	432 (234)	—	—
Chlorpromazine equivalent (excluding clozapine) dosage mean (sd) mg/day	142 (286)	—	—
Age of onset, mean (sd) years	22.5 (6)	—	—
Duration of illness, mean (sd) years	17 (8)	—	—
PANSS scores, mean (sd)			
Positive	10 (6)	—	—
Negative	15 (5)	—	—
Disorganized	8 (3)	—	—
Excitement	6 (2)	—	—
Depression	6 (3)	—	—
Total	62 (14)	—	—

*CEU* Northern and Western European ancestry, *TRS* treatment-resistant schizophrenia, *RIN* RNA integrity number, *PANSS* Positive and Negative Syndrome Scale, *mg* milligram

^a^Independent sample *t*-test

^b^Chi-square(*χ* ^2^) test

*P < 0.05

All participants were administered the Mini International Neuropsychiatric Interview (MINI)^22^ to confirm the diagnosis of schizophrenia and to rule out current or past psychiatric illness in healthy controls. Clinical symptoms were assessed using the Positive and Negative Syndrome Scale (PANSS)^23^ and scored in accordance with the consensus five-factor (i.e., positive, negative, depressed, excited, disorganized/concrete) PANSS model^24^. Tobacco, alcohol, and illicit drug use in the past 3-months was collected using a substance use questionnaire. Blood was collected after overnight fasting and processed according to standardized blood collection and processing protocol (see Supplementary Methods for more details). Clozapine plasma level was measured and current chlorpromazine equivalent dosage (except clozapine) was calculated in all patients by following standard guidelines^25,26^. The study was approved by the Melbourne Health Human Research Ethics Committee (MHREC ID 2012.069), and all participants provided written informed consent prior to participation.

#### In vitro clozapine exposure samples

Fresh frozen human PBMCs were obtained from 15 healthy donors (eight males and seven females) of Caucasian ethnicity with a mean age of 35 (sd = 13.5; range 20–54 years) from STEMCELL™ Technologies Inc. (Vancouver, British Columbia, Canada). One-third (*n* = 5) of the PBMC donors were current smokers. All the donors tested negative for HIV-1, HIV-2, Hepatitis B, and Hepatitis C. Sample size calculations showed 15 samples were sufficient to detect a large effect (Cohen’s *d* = 0.80) between exposed and unexposed conditions at *α* = 0.05 and power = 0.80.

PBMCs were isolated from peripheral blood and were supplied as vials of 100 million cells. PBMCs were seeded at a concentration of 2 million cells per well (1 × 10^6^ cells/mL) in triplicate in six-well plates and incubated in RPMI-1640 medium (Sigma-Aldrich; St. Louis, Missouri, USA) supplemented with l-glutamine (0.3 g/L) and sodium bicarbonate (2 g/L), penicillin (100units/mL), streptomycin (100 µg/mL), 10% fetal bovine serum for 24 h. PBMCs were then exposed to clozapine (Sigma-Aldrich, St. Louis, Missouri, USA) at a concentration of 1.2 µM (control wells exposed to vehicle only, see Supplementary Methods for details) and incubated at 37 °C in 5% CO_2_. Absolute ethanol was used to dissolve clozapine and media was used for dilution. The concentration of clozapine used was determined from the mean plasma concentration of clozapine found in the first 48 recruited clinical samples (1.2 µM or 384 ng/mL). Toxicity assays (CytoTox 96® Non-Radioactive Cytotoxicity Assay; Promega Corporation, Madison, Wisconsin, USA) were performed at baseline, 24 h and 7-day time points to measure the production of lactate dehydrogenase within the media (see Supplementary Fig. S1 for more details).

### SNP selection, DNA extraction, and genotyping

Fourteen *NRG1* single-nucleotide polymorphisms (SNPs) were selected based on their previously reported associations with schizophrenia (for review see refs. ^3,4^) along with 60 unlinked ancestry-informative markers (Supplementary Table S1) representing the three HapMap phase III populations (Northern/Western European, Han Chinese, and Yoruba in Nigeria)^27^. DNA extraction and quantification were performed using standard procedures described in detail in the Supplementary Methods. SNPs were genotyped at the Australian Genome Research Facility (Brisbane, Australia) with the Sequenom MassARRAY MALDI-TOF genotyping system using Sequenom iPLEX Gold chemistries according to manufacturer’s instructions (Sequenom, Inc., San Diego, CA). Two (rs113317778 and rs6150532) of the 14 *NRG1* SNPs assessed failed (0% call rate) but call rates for all remaining SNPs including the 60 ancestry markers were > 96% (Supplementary Table S1).

### RNA extraction and gene expression analysis

RNA extraction and quantification for both clinical and in vitro samples were performed using PureLink RNA Mini Kit (ThermoFisher scientific, Waltham, MA, USA) per the standard manufacturer’s instructions. Total RNA from both clinical and in vitro samples was reverse transcribed to cDNA using SuperScript® IV First-Strand Synthesis System (Invitrogen, Foster city, CA, USA) using random hexamers. cDNA (10.25 ng) was used as a template for quantitative reverse transcriptase (RT-qPCR) using master-mix and gene specific validated Taqman assays from Applied Biosystems, Foster City, California, USA. Custom designed primer and probe combinations were used for *NRG1* isoforms (type I_(Ig2)_, type II and type IV) previously investigated^9,14,28^, while inventoried assays (TaqMan®, aInvitrogen, USA) were used for all other *NRG1* isoforms (type III, Pan-*NRG1*, type I, EGFα and EGFβ) and four reference genes (beta-actin, *ACTB*; ubiquitin C, *UBC*; glyceraldehyde-3-phosphate dehydrogenase, *GAPDH*; and TATA box-binding protein, *TBP*). *NRG1* mRNA isoforms were selected based on reported associations in previous gene expression experiments using post-mortem brain tissue or peripheral blood from schizophrenia patients^9,14,15,29,30^. See Supplementary Table S2 and Fig. S2 for a list and genomic locations of each of the *NRG1* probes and primers.

Gene expression levels were determined in duplicate using FAM-MGB TaqMan® gene expression probes (Invitrogen, Foster city, CA, USA) in 192 × 24 Dynamic Arrays IFC in Fluidigm® BioMark™ HD system (South San Francisco, CA, USA) at the Monash Health Translation Precinct Medical Genomics Facility (Hudson Institute of Medical Research, Clayton, VIC, Australia). In addition, no reverse transcriptase controls and no template controls were included to rule out genomic DNA contamination and reagent contamination, respectively. Adhering to minimum information for publication of RT-qPCR (MIQE) guidelines^31^, normalized relative quantities (NRQ, i.e., 2^-ΔCt^ where Δ*C*_t_ = Ct_(candidate gene)_ - Ct _(geometric mean of reference genes)_) of each *NRG1* mRNA isoform was calculated using the geometric mean expression of two reference genes (ACTB and UBC) that did not differ between groups in either the clinical or in vitro cohorts, with the exception of the 24-hour in vitro time point for which no reference gene was stable. GAPDH and TBP were not used as reference genes because their expression differed significantly by group in both the clinical and in vitro cohorts (Supplementary Figs. S3–S5).

### Protein quantification

#### Clinical samples

Human NRG1-β1 ELISA kits (Catalog number: EHNRG1; ThermoFisher Scientific™, Life Technologies®, Waltham, MA, USA) were used to measure NRG1-β1 levels in serum according to the manufacturer’s protocol (see Supplementary Methods for details). In brief, 100 ul of serum or standard *NRG1*-β1 (0 pg/mL-20,000 pg/mL) was added to the wells in duplicate. The ELISA kits have a sensitivity of 50 pg/mL. Absorbance was measured on a SpectraMax® M3 multi-mode microplate reader (Molecular Devices, LLC; Sunnyvale, CA, USA) at 450 nm and 550 nm wavelength. The 550 nm values were subtracted from the 450 nm values to correct for optical imperfections in the microplate. A standard curve was generated for each assay by plotting mean absorbance for each standard concentration vs. the corresponding *NRG1*-β1 concentration. The standard curve (*r* ^2^ ≥ 0.99) was generated with a four-parameter logistic curve fit. The concentration of *NRG1*-β1 in the serum samples was obtained by interpolating the absorbance values using the standard curve in GraphPad Prism 6.

#### In vitro samples

The same *NRG1*-β1 ELISA kit used for the clinical samples was also used for the in vitro samples. Prior to ELISA, protein lysates were prepared from both the 24-hour clozapine exposed and control cells using RIPA buffer (Sigma-Aldrich®, Saint Louis, Missouri, USA). Due to limited baseline quantity of cells, protein lysates at 7 days were not available. The amount of total protein was quantified from each cell lysate using the ThermoScientific™ Pierce™ BCA Protein Assay Kit (ThermoFisher Scientific, MA, USA). Absorbance was measured at 562 nm using SpectraMax® M3 microplate reader. A standard curve (*r* ^2^ ≥ 0.99) was generated by plotting the absorbance value at 562 nm for each bovine serum albumin (BSA) standard vs its concentration (µg/mL). The total protein concentration of each unknown sample was determined using the standard curve. Five microgram of total cell lysate samples were mixed with appropriate amount 1x assay diluent B to be used in the ELISA system. One-hundred microliters of total cell lysate samples (0.05 µg/µL) or standard NRG1-β1 (0 pg/mL-20,000 pg/mL) was added to the wells in duplicate. Assay diluent B was used to prepare standards and served as the zero standards (0 pg/mL).

### Statistical analysis

Two-tailed tests were used for all statistical analyses. Quantile–quantile (Q–Q) plots and the Shapiro–Wilk test were used to assess normality of variable distributions. Student’s *t*-tests were used to test differences for continuous variables between schizophrenia patients and healthy controls, while chi-squared (*χ* ^2^) tests were used for categorical variables. The Benjamini and Hochberg (B–H) step-up procedure^32^ was used to adjust for multiple comparisons for all analyses. Effect sizes were calculated using the Hedges’ *g* method^33^.

#### NRG1 isoform/protein analysis

Prior to analysis, the normalized relative quantity data for all the *NRG1* isoforms and the *NRG1*-β1 data were checked for normality using Q–Q plots (Supplementary Fig. S4) and as required were log10 transformed for subsequent analysis. The log-transformed values were compared among groups using a general linear model (GLM), with the group as a fixed factor and age, gender, RNA integrity number ((RIN) (isoforms only)), and current smoking status as covariates. Despite differences in alcohol use between the schizophrenia and control groups, alcohol was not included as a covariate because it had no effect on *NRG1* isoform or protein expression (see Supplementary Table S3). For protein analysis, we used the generalized linear model, as *NRG1*-β1 levels were not normally distributed (see Supplementary Fig. S6). Outliers were identified using the Grubbs’ test for outliers and removed from further analysis.

Within the schizophrenia group, Pearson correlations between *NRG1* isoform/protein levels and symptom severity, age of onset, illness duration, current chlorpromazine equivalent dose, and clozapine plasma levels were assessed. In addition, *NRG1* isoform/protein levels between participants in positive symptom remission and non-remission were assessed using a *t*-test. Positive symptom remission was defined as a score of ≤ 3 on four PANSS items (delusions, hallucinations, grandiosity, and unusual thought content)^24^.

#### SNP and haplotype analysis

*NRG1* SNPs were mapped using the GRCh38/hg19 human genome reference assembly. Linkage disequilibrium (LD) between SNPs was examined in Haploview and haplotype blocks determined using the solid spine method^34^. For each individual, haplotypes were determined based on the best posterior probability procedure implemented in PLINK 1.07^35^. GLMs were used to explore cis-regulatory effects of *NRG1* SNPs and haplotypes on isoforms and protein expression. Each GLM included genotype/haplotype, case status, genotype/haplotype x case status as well as other relevant covariates (age, gender, RIN). Significant genotype/haplotype x case status interactions were analyzed post hoc by case status stratification analyses.

#### In vitro clozapine exposure analysis

Linear mixed models were used to determine the differences in gene expression over two-time points. In this model, the difference in transcript levels was the outcome variable and was adjusted for age, gender, and RIN. Due to non-normal distributions, Wilcoxon matched pair *t*-test was used to measure the difference in gene expression between clozapine exposed and unexposed cells at each time point.

## Results

### *NRG1* mRNA expression

**Fig. 1:**
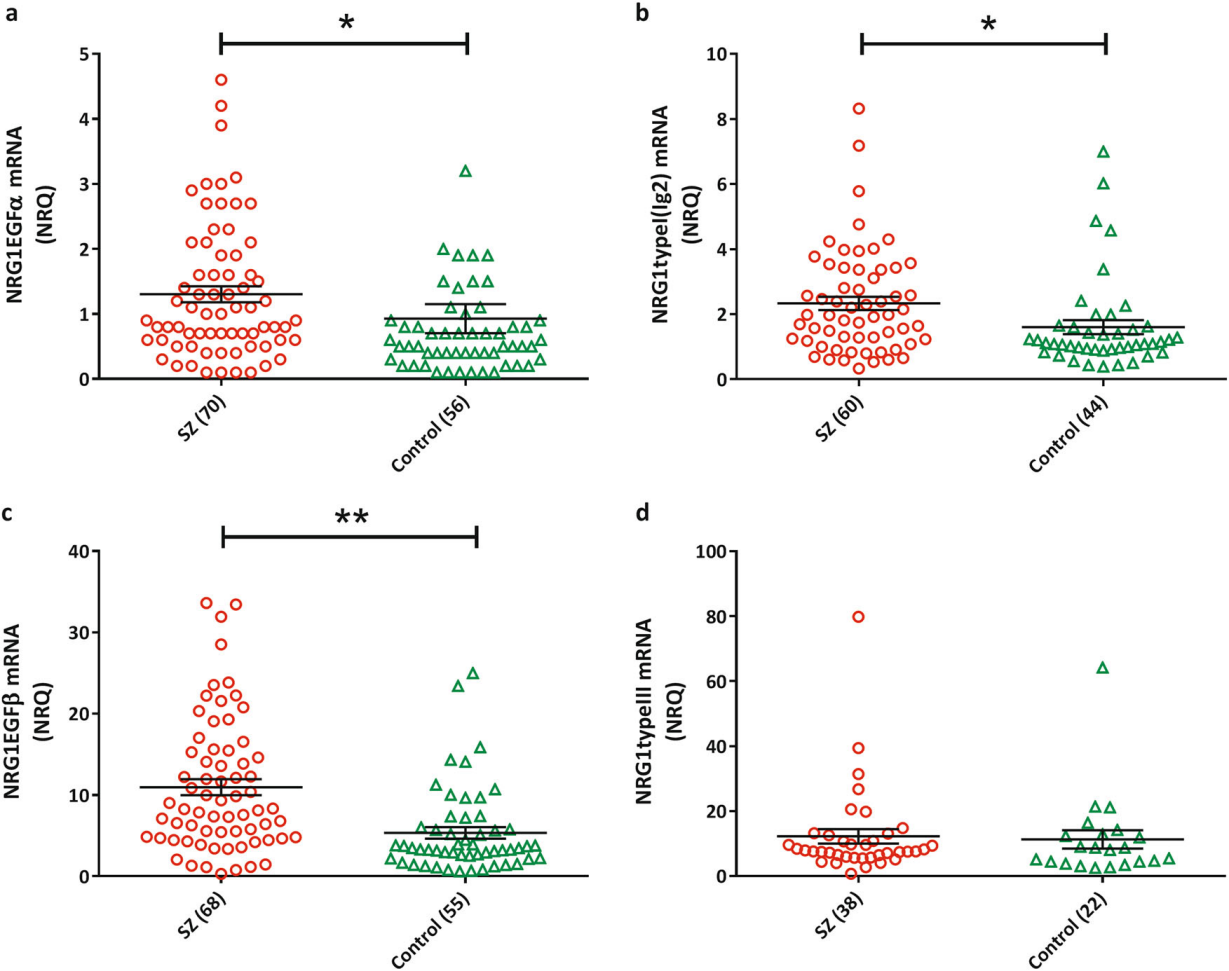
Normalized relative quantities (NRQ) of NRG1 mRNA isoforms **a** *NRG1* EGFα (schizophrenia: 1.30 ± 0.12, controls: 0.93 ± 0.22; *F*_1,125_ = 7.56, *P* = 0.0175, Hedges’ *g* = 0.33); **b** *NRG1* type I_(Ig2)_ (schizophrenia: 2.23 ± 0.21, controls: 1.61 ± 0.22; *F*_1,103_ = 6.261, *P* = 0.023, *g* = 0.70); **c** *NRG1* EGFβ (schizophrenia: 10.95 ± 0.99, controls: 7.30 ± 2.08; *F*_1,122_ = 13.14, *P* = 0.002, *g* = 0.66); and **d** *NRG1* type III (schizophrenia: 12.22 ± 2.23, controls: 11.3 ± 2.80; *F*_1,59_ = 4.23E^−10^, *P* = 1.0, *g* = 0.02). Error bars represent mean ± s.e.m. Benjamini-Hochberg adjusted *P*-values are shown. *P < 0.05 and **P < 0.01

**Fig. 2:**
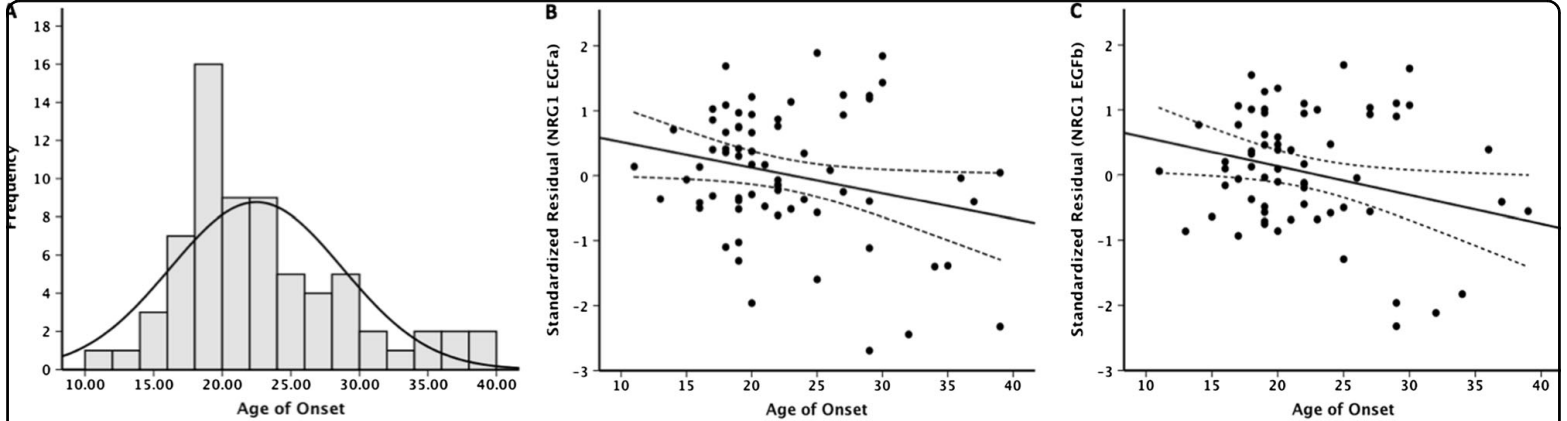
Distribution of age of onset at first diagnosis **a** and the correlation between age of onset and *NRG1* EGFα (*r* = −0.37, *P*_raw = _0.002, *P*_B-H_ = 0.02) **b** and *NRG1* EGFβ (*r* = −0.36, *P*_raw = _0.001, *P*_B-H_ = 0.02) **c** expression. *NRG1* isoform expression is represented as the standardized residual from a linear regression model after adjusting for the significant effect of age on expression. Dotted lines represented the 95% confidence intervals for the linear regression line (solid black line)

Among the eight *NRG1* mRNA isoforms interrogated, four of them (type I, type II, pan-*NRG1*, and type IV) were not detectable in more than 60% of the full cohort and so were removed from further analysis. Rates of non-detection were evenly distributed between cases and controls, with the exception of *NRG1* type II, which had a greater non-detect rate in controls (*P* = 0.00016, Supplementary Table S4). Among the remaining four *NRG1* isoforms, levels of EGFα, EGFβ, and type I_(Ig2)_ mRNA were significantly elevated and type III did not differ in schizophrenia patients compared to healthy controls after adjustment for covariates and correction for multiple testing (Fig. 1). Importantly, gene expression levels of *NRG1* isoforms were not correlated with clozapine plasma levels or chlorpromazine equivalent antipsychotic exposure (excluding clozapine) (Supplementary Table S5), which was further corroborated by our in vitro analysis that showed no difference in mRNA levels of detectable isoforms (EGFα, EGFβ, and type II) in clozapine exposed compared to unexposed PBMCs (Supplementary Fig. S7). Furthermore, within the patients with schizophrenia significant negative correlations between age of onset and *NRG1* EGFα (*r* = −0.37, *P*_raw = _0.002, *P*_B-H_ = 0.02) and EGFβ (*r* = −0.36, *P*_raw = _0.001, *P*_B-H_ = 0.02) expression were detected (Fig. 2, Supplementary Table S5). No significant correlations were observed between *NRG1* isoforms and duration of illness after adjustment for multiple testing, although a trend-level negative correlation was found between *NRG1* type III expression and duration of illness (*r* = −0.36, *P*_raw = _0.027, *P*_B-H_ = 0.167).

### NRG1 protein expression

**Fig. 3:**
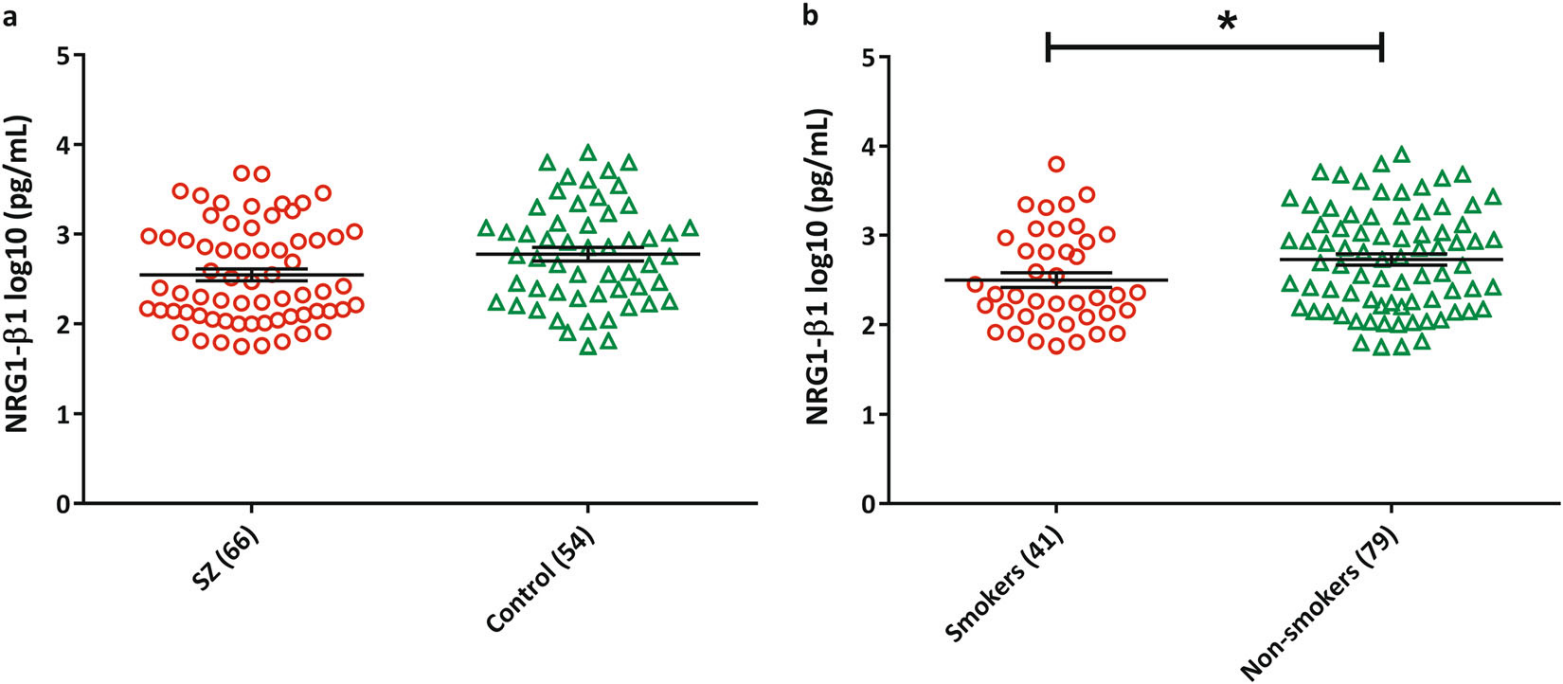
NRG1-β1 protein expression between **a** schizophrenia and controls (schizophrenia: 2.55 ± 0.067 pg/mL, controls: 2.78 ± 0.077 pg/mL; *P*_unadjusted_ = 0.019, *P*_adjusted for smoking_ = 0.050, Hedges’ *g* = 0.42) and **b** current smokers and non-smokers (smokers: 2.49 ± 0.083 pg/mL, non-smokers: 2.73 ± 0.064 pg/mL; *t* = −2.153, df = 118, *P* = 0.033, *g* = 0.43). Error bars represent mean ± s.e.m. *P < 0.05

In contrast to the increase in NRG1 mRNAs, we found that NRG1-β1 protein levels were lower in schizophrenia patients compared to healthy controls (*P* = 0.019) but after adjustment for smoking status, this finding was attenuated (*P* = 0.050; Fig. 3a). Current smokers had lower NRG1-β1 protein levels compared to non-smokers in the full cohort (*P* = 0.033, Fig. 3b) and schizophrenia participants were more likely to be current smokers as compared to controls (46.5% vs. 21.1%, *P* = 0.002, Table 1). However, clozapine plasma levels were not associated with NRG1-β1 expression (*r* = −0.023, *P* = 0.85), and there was no difference in NRG1-β1 protein levels between clozapine exposed and unexposed cells (exposed: median 2.31 log10 pg/mL, unexposed: median 2.2 log10 pg/mL; *P* = 0.191; Supplementary Fig. S8). We found no association between NRG1-β1 protein levels and chlorpromazine equivalent antipsychotic exposure, age of onset, or illness duration (Supplementary Table S5).

### NRG1 isoforms/protein expression and symptomatology

Significant negative correlations between *NRG1* mRNA isoform EGFα expression and depression severity score (*r* = −0.241, *P*_raw = _0.045, *P*_B-H = _0.270) as well as type III expression and positive symptom severity score (*r* = −0.377, *P*_raw_ = 0.020, *P*_B-H_ = 0.120) were observed but did not survive correction for multiple comparisons (Supplementary Table S6). An exploratory examination of schizophrenia patients in positive symptom remission vs. non-remission revealed no statistically significant differences in levels of any of the *NRG1* isoforms or NRG1-β1 serum protein after correction for multiple comparisons, although a trend (*P*_raw_ = 0.013, *P*_B-H_ = 0.065) toward greater *NRG1* type III expression in remitters vs. non-remitters was observed (Supplementary Table S7).

### Genotype and haplotype effects on NRG1 isoforms/protein expression

LD analysis revealed two haplotype blocks among the 12 successfully genotyped SNPs (Supplementary Fig. S9). Block 1 contained four of the Icelandic schizophrenia-risk haplotype (Hap_ICE_) SNPs (rs73235619, rs35753505, rs62510682, rs6994992) along with two other SNPs (rs4281084 & rs7014762) in the 5'-region and Block 2 included four SNPs (rs3924999, rs2439272, rs2954041, rs74942016) in the 3'-region of *NRG1*. Examination of these haplotypes, as well as each SNP, independently revealed several nominal *NRG1* isoforms and protein expression quantitative trait loci but none survived correction for multiple comparisons (Supplementary Table S8).

## Discussion

Among the four detectable *NRG1* isoforms in whole blood three (EGFα, EGFβ, and type I_(Ig2)_) were elevated and one (type III) did not differ between clozapine-treated schizophrenia patients and healthy controls. Importantly, we could not attribute these overall increases in *NRG1* mRNA levels to demographic characteristics and did not find a correlation with clozapine blood levels, suggesting that elevated *NRG1* mRNA in whole blood may not be a direct consequence of age, sex, smoking, or exposure to clozapine; the latter supported by our in vitro experiments. However, age of illness onset was negatively correlated with expression of *NRG1* EGFα and EGFβ containing trancripts, suggesting increased expression of these isoforms are assocated with an earlier age of illness onset.

To our knowledge, we are the first to report elevated levels of *NRG1* EGFα, EGFβ, and type I_(Ig2)_ in schizophrenia, specifically in those with treatment-resistant schizophrenia. Previous peripheral expression studies have not measured these isoforms^15–17,36^, although a previous study examining peripheral expression of two other *NRG1* isoforms (ndf43a and ndf43b) covered by the *NRG1* EGFβ probe reported no difference between schizophrenia and control participants^15^. Furthermore, one post-mortem study, which specifically measured the EGF domain containing mRNAs reported no difference in *NRG1* EGFβ levels and was unable to reliably detect *NRG1* EGFα or type I_(Ig2)_ in the dorsolateral prefrontal cortex of schizophrenia and control participants^14^. This suggests our findings, if extended, may contribute to a unique *NRG1* mRNA alteration (signature) in the blood of individuals with schizophrenia or more specifically treatment-resistant schizophrenia. However, in the current study we were unable to compare individuals with and without treatment-resistant schizophrenia and as such the ability of these *NRG1* isoforms to identify treatment-resistant schizophrenia patients remains to be confirmed.

We also found a novel and robust negative correlation between age of onset and expression of EGFα and EGFβ isoform levels, suggesting elevated levels of these isoforms were more frequently detected in those with an earlier age of illness onset. Interestingly, a previous post-mortem brain study^14^, showed that brain abundant *NRG1* type III expression was negatively correlated with age of onset. These findings across two different cohorts and from two different cell populations, suggest a relationship between age of onset and *NRG1* gene expression in both brain and blood such that higher gene expression of *NRG1* may accelerate or serve to precipitate transition to full blown symptoms. While, these studies suggest that the distinct *NRG1* isoforms may monitor clinically meaningful events in blood as compared to brain, and suggest the possibility that blood measures of *NRG1* could serve as surrogate markers for *NRG1* in brain. Future longitudinal studies are needed to explore whether elevated *NRG1* gene expression is a precipitating factor and/or a consequence of an earlier age of onset.

Our analyses showed no difference in the expression of *NRG1* type III between schizophrenia patients and controls, which do not concur with a previous study that reported increased expression of *NRG1* type III in peripheral leukocytes of schizophrenia patients^15^. *NRG1* type III is the most abundant of all *NRG1* isoforms in the human brain and increased expression of this isoform was found to be associated with genetic variation in the *NRG1* Hap_ICE_ region^9,14^. However, we were unable to replicate the increase *NRG1* type III in whole blood. We did however, find trend-level negative correlations between *NRG1* type III expression and duration of illness and positive symptom severity as well as elevated expression in remitters, providing preliminary evidence that downregulation of this isoform in blood may occur with disease progression but this in turn is associated with greater positive symptom severity and lower likelihood of achieving positive symptom remission. To our knowledge no other blood-based study of *NRG1* type III expression has examined these associations and as such it is not clear if they are unique to treatment-resistant schizophrenia or are generalizable to all those with a schizophrenia diagnosis.

We could not detect *NRG1* type IV mRNA in any sample and pan-*NRG1*, type I, and type II mRNAs were not detectable in greater than 60% of our cohort. Our failure to detect *NRG1* type IV and type I are in alignment with a previous study that failed to detect these isoforms in immortalized lymphocytes^36^. However, pan-*NRG1* was shown to be decreased in peripheral lymphocytes^16,17^ and type II β3 increased in peripheral leukocytes^15^, suggesting detection of these isoforms in the periphery may depend on the cell populations examined. We did observe, however, a significantly lower frequency of type II non-detects among our schizophrenia group compared to controls (55% vs. 86%, *P* < 0.01, Supplementary Table S4), indicating that there may be elevated type II expression in schizophrenia. Still, our low detection of pan-*NRG1* was unexpected given that the probes for this transcript targeted both the Ig1 and Ig2 regions of the *NRG1* gene, which all isoforms we measured contain (see Supplementary Fig. S2 for the regions of amplification for each *NRG1* isofrom). However, for all the isoforms with low detection the probes we used targeted the Ig1 region, suggesting this region of *NRG1* may be downregulated in whole blood and resulted in lower amplification. Although, these probes and primers have been succesfully employed by our group in postmortem human brain, to our knowledge this is the first time they have been used in whole blood. Future studies using whole blood should consider alternative probes for these *NRG1* isoforms.

In contrast to our mRNA findings, NRG1-β1 protein abundance was lower in clozapine-treated schizophrenia patients relative to controls. This finding did not appear to be related to demographic characteristics, genetic variation, or clozapine dose or clozapine blood levels (confirmed by our in vitro clozapine exposure experiment) but was attenuated after adjustment for smoking status. To our knowledge, previous studies measuring peripheral or central NRG1 protein levels have not accounted for smoking status as a potential confound. Our results suggest smokers, regardless of case status, have lower peripheral NRG1-β1 protein levels than non-smokers. Given that smoking prevalence rates are known to be significantly higher among individuals with schizophrenia compared to the general population^37^, it is possible that previously reported differences in NRG1 protein levels between schizophrenia and control participants may have also been confounded by smoking. We are aware of two previous studies that have examined peripheral NRG1 protein levels in schizophrenia. The first reported lower Ig-NRG1 levels in serum^38^ and the second reported lower NRG1-β1 protein in plasma from schizophrenia patients^18^. However, neither study adjusted for smoking status in their analyses. It is not clear if smoking status would have similar effects on brain NRG1 protein levels reported in post-mortem studies^8,11,39^, as none of these studies examined this potential effect and it remains uncertain whether NRG1 protein levels in the brain concur with levels in the periphery. Nevertheless, our findings provide reasonable evidence for inclusion of smoking as a potential confound when measuring and interpreting NRG1 protein levels in groups with known differences in smoking prevalence and support future pre-clinical experiments assessing the effect of smoking on NRG1 protein levels in blood and brain.

Several limitations should be noted. First, the size of the cohort only allowed for detection of moderate to large differences in mRNA and protein abundances between groups. Second, the study employed a cross-sectional design, inhibiting examination of temporal expression patterns and how these patterns map on to clinical trajectories. Third, measurement of mRNA and protein expression occurred in whole blood and serum, respectively. Although both of these tissues are clinically accessible and commonly used in biomarker research, it is not fully clear how well our findings will generalize to other peripheral (e.g., plasma, lymphocytes) or central (e.g., brain) tissues despite some support for their applicability in schizophrenia^20^. In addition, the generalizability of our findings beyond those with treatment-resistant schizophrenia is not clear. Future studies comparing *NRG1* gene and protein expression between treatment-resistant and non-resistance schizophrenia patients, including treatment-naive patients, are warranted. Finally, our in vitro clozapine exposure experiments examined a single clozapine concentration (1.2 µM) guided by pilot data from our study population. A previous study used higher clozapine concentrations (2 µM) for three weeks in cultured post-mortem human fetal brain tissue and showed an upregulation of *NRG1* protein^40^. As such, future work with PBMCs should examine multiple concentrations that reflect the range of clozapine blood levels observed in the clinic. Future in vitro clozapine experiments with PBMCs should also screen a greater number of genes to identify more suitable references, particularly genes that are stable during acute clozapine exposure.

Despite these limitations, the current study represents the first comprehensive investigation of *NRG1* isoforms and protein expression in whole blood of clozapine-treated schizophrenia patients. In general, our results support the notion posed by previous peripheral blood and post-mortem brain studies that *NRG1* transcription is dysregulated in schizophrenia and perhaps more specifically treatment-resistant schizophrenia. However, we have also expanded on this by showing *NRG1* mRNA isoforms EGFα, EGFβ, and type I_(Ig2)_ are elevated in whole blood of clozapine-treated schizophrenia patients. Our findings further suggest that *NRG1* expression is associated with age of onset, particularly *NRG1* EGFα and EGFβ isoforms and that *NRG1* type III expression may vary by disease progression. As such our results suggest that *NRG1* overexpression may not be restricted to the brain of those with schizophrenia, and blood-based *NRG1* transcription may serve, in part, as a suitable biomarker for schizophrenia and perhaps treatment-resistant schizophrenia.

## Electronic supplementary material

Supplementary Materials
