# Supplementary material for: Elevated peripheral expression of neuregulin-1 (NRG1) mRNA isoforms in clozapine-treated schizophrenia patients
Source: Transl Psychiatry. 2017 Dec 11;7:1280. doi: 10.1038/s41398-017-0041-2 (PMC5802529; doi:10.1038/s41398-017-0041-2)
Supplement: Supplementary file 1 — Supplementary Materials [file 41398_2017_41_MOESM1_ESM.docx]

**SUPPLEMENTARY MATERIAL**

**Elevated peripheral expression of neuregulin-1 (NRG1) mRNA isoforms in clozapine-treated schizophrenia patients**

| **Table of contents** | **Page no.** |
| --- | --- |
| **Supplementary Methods** | **2-8** |
|  |  |

**Supplementary Figures**

| **Figure S1** | LDH toxicity assay results at 24 hour and 7 days post clozapine exposure | **9** |
| --- | --- | --- |
| **Figure S2** | *NRG1* mRNA probes and their region of amplification on the *NRG1* gene | **10** |
| **Figure S3** | Expression of reference genes between schizophrenia patients vs healthy controls | **11** |
| **Figure S4** | Expression of reference genes after 7-days clozapine exposure | **12** |
| **Figure S5** | Expression of reference genes after 24 hour clozapine exposure | **13** |
| **Figure S6** | Quantile-quantile plots of log10 transformed NRQ values for NRG1 isoforms & protein | **14** |
| **Figure S7** | Expression of detectable NRG1 isoforms after 7 days clozapine exposure. | **15** |
| **Figure S8** | Expression of NRG1-β1 after 24 hour clozapine exposure | **16** |
| **Figure S9** | Linkage disequilibrium (D’) between SNPs in the *NRG1* gene | **17** |

**Supplementary Tables**

| **Table S1.** | NRG1 SNPs with ancestry informative markers and their corresponding call rates | **18** |
| --- | --- | --- |
| **Table S2.** | TaqMan probes and primer sequence for quantification and normalization of different NRG1 isoform expression | **19** |
| **Table S3** | Normalized relative quantities (NRQ) of detectable *NRG1* mRNA isoforms and NRG1-β1 by alcohol use. | **20** |
| **Table S4.** | Number of non-detects n (%) for all *NRG1* isoforms assessed | **21** |
| **Table S5.** | Pearson’s correlation between different NRG1 isoforms &NRG1-β1 with clozapine plasma level and chlorpromazine equivalent antipsychotic exposure | **22** |
| **Table S6.** | Pearson’s correlation between NRG1 isoforms and NRG1-β1 protein level with PANSS positive, negative, disorganized, excited, depression score and total score. | **23** |
| **Table S7.** | Normalized relative quantities (NRQ) of detectable *NRG1* mRNA isoforms and NRG1-β1 by remission status. | **24** |
| **Table S8** | Expression quantitative trait loci (eQTL) analysis of putative NRG1 SNPs with NRG1 isoforms and NRG1-β1 protein expression | **25** |
| **References** |  | **26** |

**Supplementary methods**

*Blood sampling method*

Blood was collected after overnight fasting and processed according to the Australian Imaging, Biomarker and Lifestyle (AIBL) blood collection and processing protocol. A trained nurse or scientist undertook the blood draw at existing facilities within the local hospital and pathology services. The blood sample was then transported to the AIBL laboratory at the Mental Health Research Institute for further processing. Briefly, blood samples were collected in EDTA tubes (for DNA), PAXgene® Blood RNA tubes (for mRNA) and in SST tube (for the collection of serum). Blood processing was done within 2 hours of venipuncture under sterile conditions. The gel serum tubes were allowed to clot before processing (at least 20 minutes following venipuncture). After the tubes were filled with blood, they were inverted 8-10 times for mixing. They were kept at ambient temperature until processed. The PAXgene tubes were allowed to stand upright at room temperature for 24 hours in the dark and then overnight at -20°C. The EDTA and SST tubes were centrifuged for 15 minutes at 1500 x g at 20°C. The serum samples were collected in Nunc™ Coded Cryobank Vial Systems (ThermoFisher Scientific™, Waltham, MA, USA). All the tubes were stored at -80°C until further processing.

*Cell culture method*

The clozapine used in this experiment was obtained from Sigma-Aldrich (St. Louis, Missouri, USA). The concentration of clozapine used in this experiment was determined from the mean plasma concentration of clozapine found in the cohort of treatment-resistant schizophrenia patients (1.2µM or 384ng/mL). Clozapine induces PBMC death at higher concentrations (5x10^-6^ to 2.5x10^-5^ M) dosage ^1^. In spite of this LDH toxicity assay was performed at baseline, 24 hours and 7 day time periods.

PBMCs were incubated in RPMI-1640 medium (Sigma-Aldrich; St. Louis, Missouri, USA) supplemented with L-glutamine (0.3g/L) and sodium bicarbonate (2g/L), penicillin (100units/mL), streptomycin (100µg/mL), 10% fetal bovine serum and 1.2µM of clozapine at 37°C in 5% CO_2_. They were seeded at a concentration of 2 million cells per well (1x10^6^ cells/mL) in triplicate in six-well plates and treated with 1.2µM of clozapine for 24 hours and 7 days. Absolute ethanol was used to prepare 10mM clozapine solution and it was diluted with media to prepare 0.48mM clozapine solution. From this 5µL was added to each well so that each well is exposed with 1.2µM of clozapine. Each control well was exposed with media only. Cells were exposed to clozapine for 24 hours and 7 days. Total RNA was extracted at both time points but protein lysates were extracted at 24-hour time point only. A separate six-well plate with 2 million cells per well (1x10^6^ cells/mL) was set up for DNA extraction.

*DNA extraction and quantification*

*Clinical samples.* DNA was recovered from approx. 2 ml EDTA-anti-coagulated blood samples using the “NucleoSpin® Blood L” silica matrix binding system (Macherey-Nagel GmbH & Co. KG, 52355 Düren, Germany) system according to manufacturer’s instructions. DNA eluates were quantified by fluorimetry (QuantiFluor™ dsDNA System; Promega Corporation, Madison, Wisconsin, USA) in conjunction with a Gemini™ Spectramax3 microplate reader (Molecular Devices, LLC; Sunnyvale, CA, USA). DNA stocks were adjusted to a working concentration of between 10 and 50 ng ul-1 for subsequent genotyping. Concentration and purity of DNA were checked by NanoDrop 2000 UV-Vis Spectrophotometer (Thermoscientific®, MA, USA).

*In-vitro samples.* Genomic DNA was extracted from the cultured cells of each individual by AccuPrep® Genomic DNA Extraction Kit from Bioneer Corporation® (Daejeon, Republic of Korea) using the standard protocol for cultured cells. Concentration and purity of DNA were checked by NanoDrop 2000 UV-Vis Spectrophotometer (Thermoscientific®, MA, USA).

*RNA extraction from clinical cohort and quality control*

For RNA extraction blood was collected in PAXgene® Blood RNA tubes (Qiagen, Hilden, Germany). Intracellular RNA from whole blood was extracted using PAXgene® Blood RNA kit using “The Manual PAXgene Blood RNA procedure” ([www.qiagen.com/at/resources/resourcedetail?id=6e32307d-7e54-4767-8f25-4d9e6b9e0d15&lang=en](http://www.qiagen.com/at/resources/resourcedetail?id=6e32307d-7e54-4767-8f25-4d9e6b9e0d15&lang=en)). The quality of extracted RNA was checked for RIN and concentration using the Agilent® RNA 6000 Nano kit on the Agilent® 2100 Bioanalyzer system (Agilent Technologies, Santa Clara, CA, USA). The lowest RIN was found to be 6.6 for one sample but for rest of samples, it was above 7 (range 7.7 – 9.3).

*RNA extraction from cultured PBMCs and quality control*

Total RNA was extracted from 24 hours and 7 day time periods from both clozapine exposed and control cells using PureLink™ RNA Mini Kit (Life technologies®, ThermoFisher Scientific™, Waltham, MA, USA) using a standard protocol. Briefly, cells were lysed with using lysis buffer containing 1% 2-mercaptoethanol. After the pellets were dispersed and cells appear lysed they were homogenized by passing the lysate 5-10 times through an 18-gauzge syringe needle. Next, RNA purification was performed using wash buffer I and II and RNA was eluted in 30µL of RNase-free water. All the RNA tubes were stored at -80°C until further use. The quality of extracted RNA was checked for RIN and concentration using the Agilent® RNA ScreenTape assay with the Agilent 2200 TapeStation system (Agilent Technologies, Santa Clara, CA, USA).

*Reverse Transcription of mRNA*

After extraction, RNA was reverse transcribed to cDNA. Then, cDNA was used as a template for RT-qPCR using master-mix and gene specific validated Taqman assays from Applied Biosystems, Foster City, California, USA. For cDNA synthesis, total RNA (200 ng) was denatured for 5 min at 65°C, and then reverse transcribed using the SuperScript® IV First-Strand cDNA Synthesis Kit (Invitrogen, Carlsbad, California, USA) in a 20μL reaction volume containing 1× Reverse Transcription SSIV buffer, 10mM dNTP mixture, 50µM random hexamers, 100mM DTT, 40 U/µl RNase OUT™ Recombinant RNase Inhibitor and 200 U/µl SuperScript® IV Reverse Transcriptase. The reaction (40 cycles) will be incubated at 23°C for 10 min, followed by 55°C for 10 min, 80°C for 10 min. To remove RNA, 1 µl of *E.coli* RNase H was added to each reaction mixture and incubated at 37°C for 20min and finally held at 4°C. All cDNA samples were stored at -20°C until qPCR analysis. In each plate, one no template control and one RT negative were used for quality control purposes.

*Real-Time Quantitative Polymerase Chain Reaction (RT-qPCR)*

Gene expression was performed using FAM-MGB TaqMan® gene expression probes (Invitrogen, Foster city, CA, USA) in 192.24 Dynamic Arrays IFC in Fluidigm® BioMark™ HD system (South San Francisco, CA, USA) at the MHTP Medical Genomics Facility (Monash Health Translation Precinct, Hudson Institute of Medical Research, Clayton, VIC, Australia). All the samples were run in duplicates in two independent 192.24 BioMark IFC arrays to ensure there was no technical variability. Prior to gene expression, quality control was performed. All the samples qualified except one and that was excluded from the further experiment. The RT negatives showed a very high Ct value indication absence or low genomic DNA contamination and the ‘no template’ control did not show any amplification.

The FAM-MGB, TaqMan gene expression assays are provided as 20x forward and reverse primer and probe mixes. Each primer is at a concentration of 18µM and the probe is at a concentration of 4µM. The TaqMan assays were selected from the Single Cell Genomics Taqman Library at the Single Cell Genomics Centre (MHTP Medical Genomics Facility, Monash Health Translation Precinct, Clayton, VIC, Australia). Custom designed primer and probe combinations were used for specific NRG1 isoforms previously investigated ^2-4^, while inventoried assays (TaqMan®, Invitrogen, Foster city, CA, USA) were used for all other NRG1 isoforms and housekeeping genes. Supplementary table (2) contains the list of the probes and primers.

Pre-amplification was done to increase the number of copies of each gene to detectable levels as detailed in Gene Expression Preamp with Fluidigm® Preamp Master Mix and TaqMan® Assays Quick Reference PN 100-5876B1. To reduce bias, the pre-amplification procedure takes probes and primers of all genes of interest and makes a probe-primer pool and then all the samples get the equal amount of the mixture for amplification. Taqman assays were firstly pooled by combining 4μL of each of the 24 20X TaqMan assays and 304μL C1 DNA suspension buffer for a final volume of 400μL. The final concentration of each assay was 0.2X (180nM).

Pre-amplification allows multiplex amplification. 3.75μL of Sample Pre-Mix (Life Technologies TaqMan® PreAmp Master Mix and Pooled Taqman assays) was combined with 1.25μL of each of the cDNA samples, RT-negative samples, and ‘no template’ water controls for a final reaction volume of 5μL per sample. An additional no template control (by the gene expression facility) was also included and all samples were pre-amplified for 14 cycles. Following pre-amplification, reaction products were diluted 1:5 by adding 20μL C1 DNA suspension buffer to the final 5μL reaction volume for a total volume of 25μL.

Assays and Samples were combined in a 192.24 Dynamic array IFC according to Fluidigm® 192.24 Real-Time PCR Workflow Quick Reference PN 100-6170. Briefly, 3μL of each assay at a final concentration of 10X was added to each assay inlet port and 3μL of diluted sample to each sample inlet port according to the Chip Pipetting Map. For unused sample inlets, 2.2uL of sample premix and 1.8uL of water per inlet were used. The data were analyzed with Fluidigm Real-Time PCR analysis software (V4.1.1).

Normalized relative quantities of different NRG1 isoforms (mRNA) were calculated relative to the geometric mean of two reference genes, beta actin (ACTB) and ubiquitin-c (UBC). The NRG1 isoforms and reference genes were selected based on a previously reported gene expression experiment conducted on post-mortem brain tissue in schizophrenia patients and controls ^5^ The relative quantities of ACTB and UBC were not significantly different between the groups in both clinical cohort and in-vitro experiment. Two of the four reference genes (GAPDH and TBP) were found to significantly different in the clinical cohort and therefore was not used as reference genes for normalization.

*Quantitation of total protein*

The amount of total protein was quantified from each cell lysate using the ThermoScientific™ Pierce™ BCA Protein Assay Kit (ThermoFisher Scientific, MA, USA). A set of diluted protein (BSA) standards (0-1000µg/mL) were prepared using the supplied albumin standard ampule (2mg/mL). The total cell lysate samples were prepared in 1:20 dilution with Milli-Q® ultrapure water (Merck Millipore™, Massachusetts, USA). BCA working reagent was prepared by diluting copper (II) sulfate solution in bicinchoninic acid (BCA) solution (1:50). 25µL standards and diluted cell lysate protein samples were mixed with 200µL of BCA working reagent (sample to working reagent ratio=1:8) in a clear flat bottom 96-well plate in triplicates. The plate was incubated at 37°C for 30 mins. Absorbance was measured at 562nm using SpectraMax® M3 microplate reader (Molecular Devices, LLC; Sunnyvale, CA, USA). A standard curve was generated by plotting the absorbance value at 562nm for each BSA standard vs its concentration (µg/mL). The total protein concentration of each unknown sample was determined using the standard curve.

*ELISA for clinical cohort*

Human NRG1-β1 ELISA kits (Catalog number: EHNRG1; ThermoFisher Scientific™, Life Technologies®, Waltham, MA, USA) were used to measure NRG1-β1 levels in serum according to manufacturer protocol. The kit comes with an ELISA plate pre-coated with the anti-human NRG1β1 antibody (NRG1 antibody 147705, Invitrogen®, ThermoFisher Scientific™, Waltham, MA, USA) and other components to perform the assay. The serum samples along with the reagents were thawed at room temperature before use in the assay. In brief, 100ul of serum and standard NRG1-β1 (0 pg/ml-20,000 pg/ml) was added to the wells in duplicate. Assay diluent A was used to prepare standards and it serves as the zero standards (0 pg/ml). The plate was incubated overnight at 4°C with gentle shaking. After four washing, 100ul of 1X prepared Biotinylated Antibody reagent was added to the wells and incubated for 1 hour at room temperature. Next, Streptavidin-HRP reagents (100ul) were added to each well followed by incubation for 45 minutes at room temperature. The plate was again washed four times and 100ul of TMB substrate solutions was loaded to each well and incubated for 30 minutes at room temperature in the dark with gentle shaking. The reactions were stopped by adding 50ul of Stop solution to each well. Absorbance was measured on a SpectraMax® M3 multi-mode microplate reader (Molecular Devices, LLC; Sunnyvale, CA, USA) at 450nm and 550nm wavelength. The 550nm values were subtracted from the 450nm values to correct for optical imperfections in the microplate. A standard curve was generated for each assay by plotting mean absorbance for each standard concentration versus the corresponding NRG1-β1 concentration. The standard curve was generated with a four-parameter logistic curve fit. The concentration of NRG1-β1 in the serum samples was obtained by interpolating the absorbance values using the standard curve in GraphPad Prism 6. According to the manufacturer, this ELISA kit has <10% of Intra-assay and <12% of Inter-assay coefficients of variation and has no cross-reactivity with BDNF, BLC, ENA-78, FGF- 4, IL-1 alpha, IL-1 beta, IL-2, IL-3, IL-4, IL-5, IL-6, IL-7, IL-8, IL-9, IL-11, IL-12 p70, IL-12 p40, IL-13, IL-15, IL-309, IP-10, G-CSF, GM-CSF, IFN-gamma, Leptin (OB), MCP-1, MCP-2, MCP-3, MDC, MIP-1 alpha, MIP-1 beta, MIP-1 delta, PARC, PDGF, RANTES, SCF, TARC, TGF-beta, TIMP-1, TIMP-2, TNF-alpha, TNF-beta, TPO, VEGF.

**
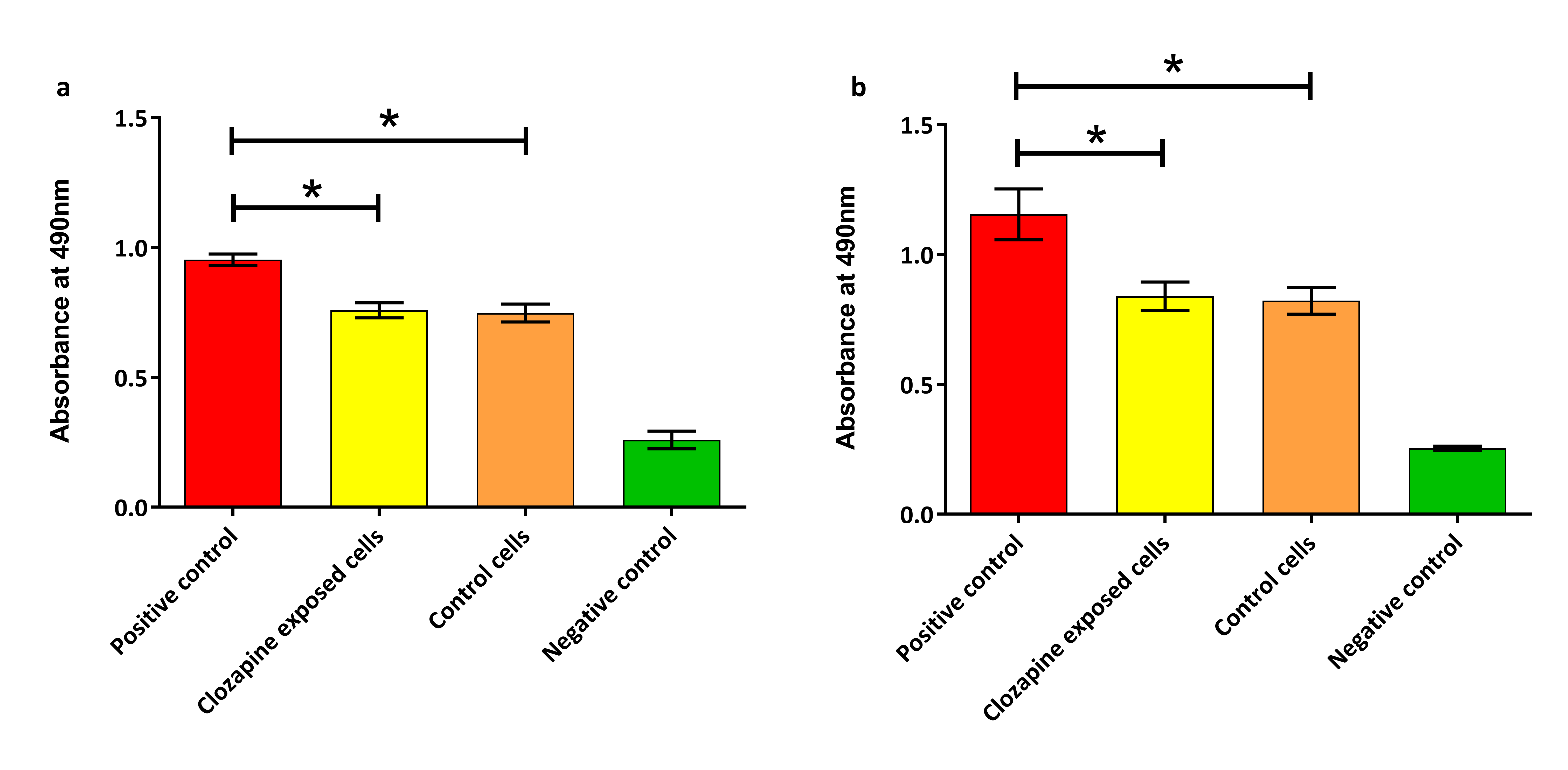
**

**Supplementary Figure S1:** LDH toxicity assay results in cultured PBMCs, (a) 24 hour post exposure and (b) 7-days post exposure. *P=0.001


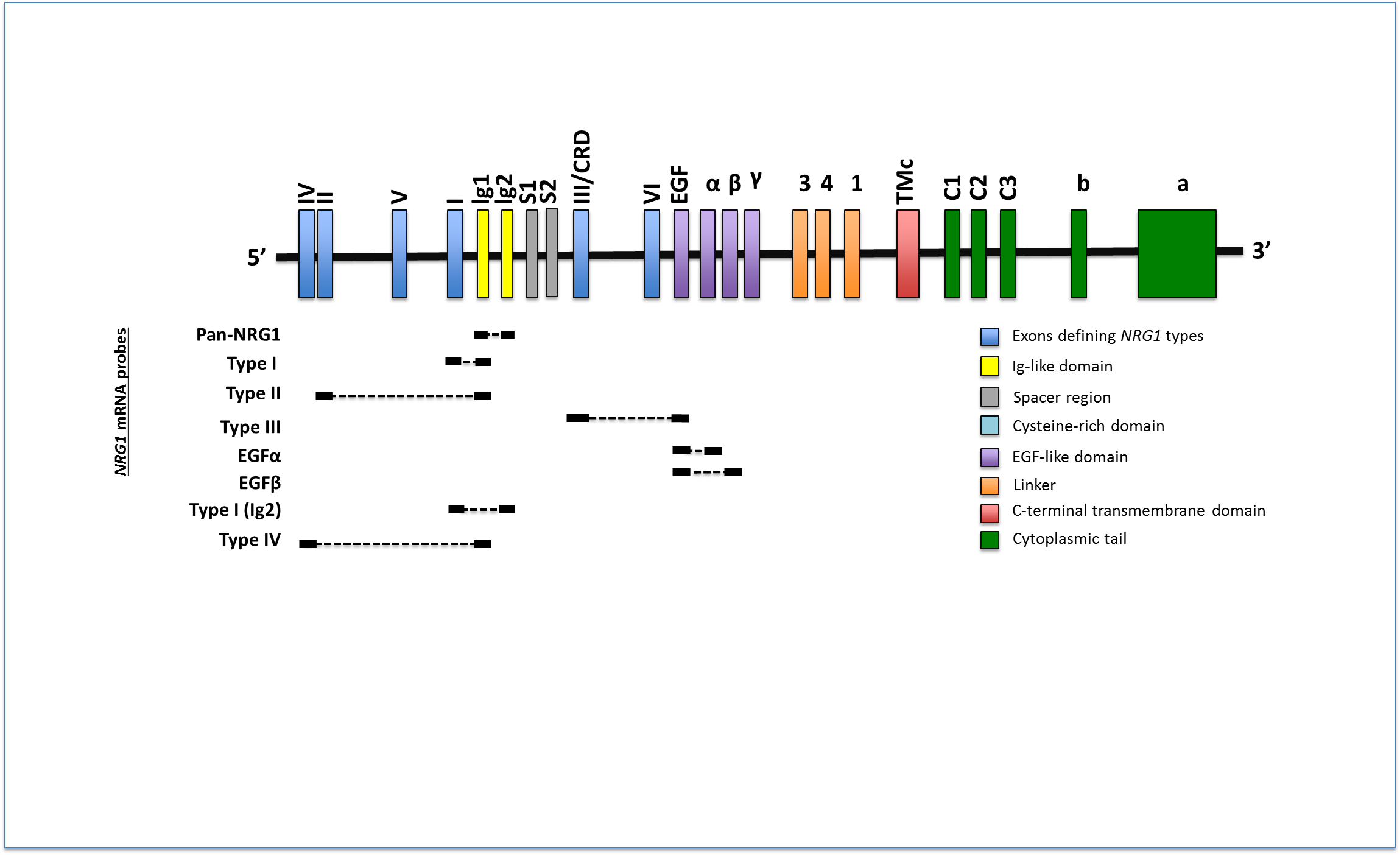


**Supplementary Figure S2:** *NRG1* mRNA probes and their region of amplification on the *NRG1* gene.


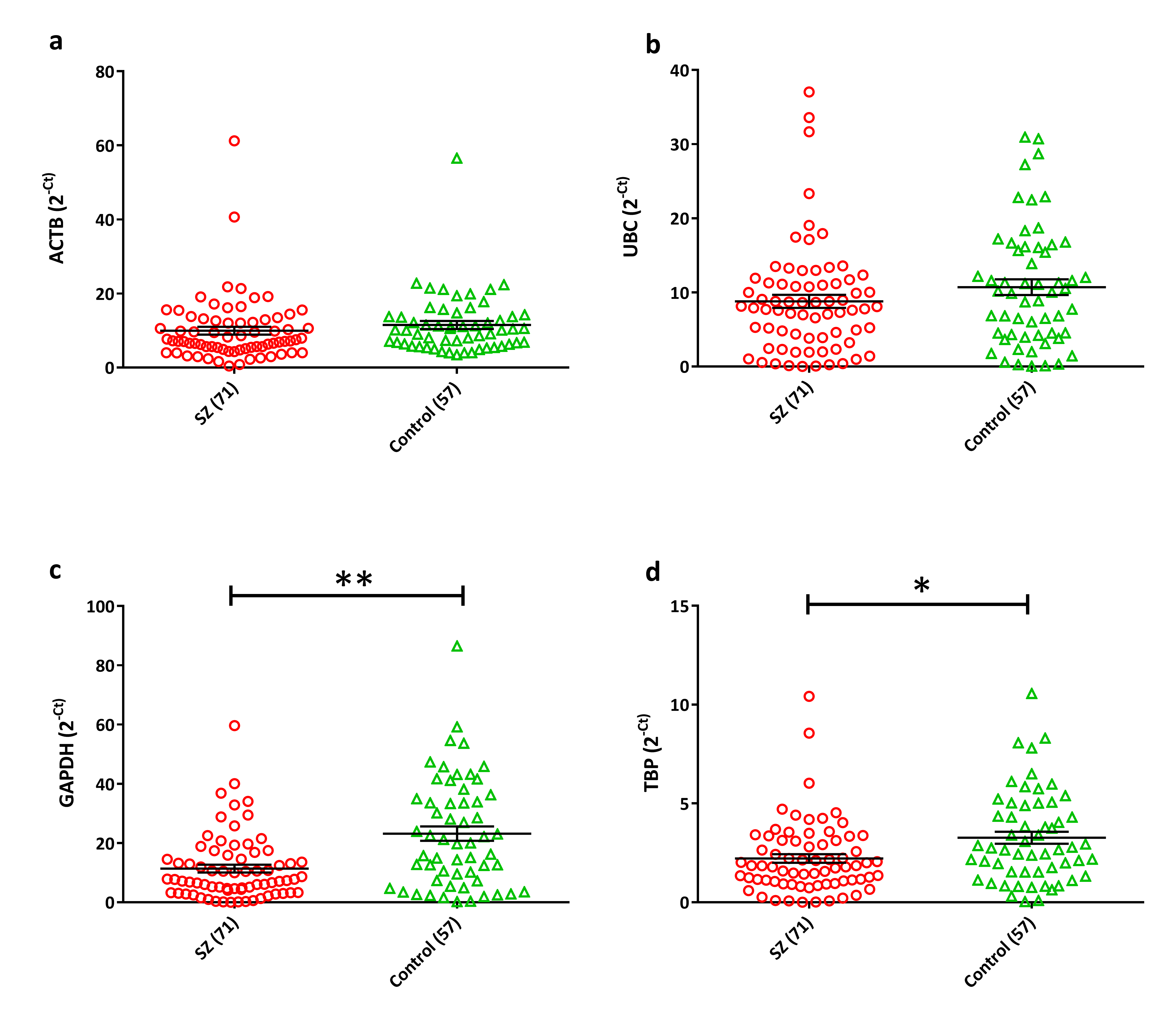


**Supplementary Figure S3**: Expression of reference genes in schizophrenia patients vs healthy controls; (**a**) ACTB (t=-1.027, df=126, P=0.306), (**b**) UBC (t=0.170, df=126, P=0.163), (**c**) GAPDH (t=-4.259, df=87.383, P=0.000052), (**d**) TBP (t=-2.810, df=105.563, P=0.006). Error bars represent mean ± s.e.m. *P<0.05, **P<0.001.


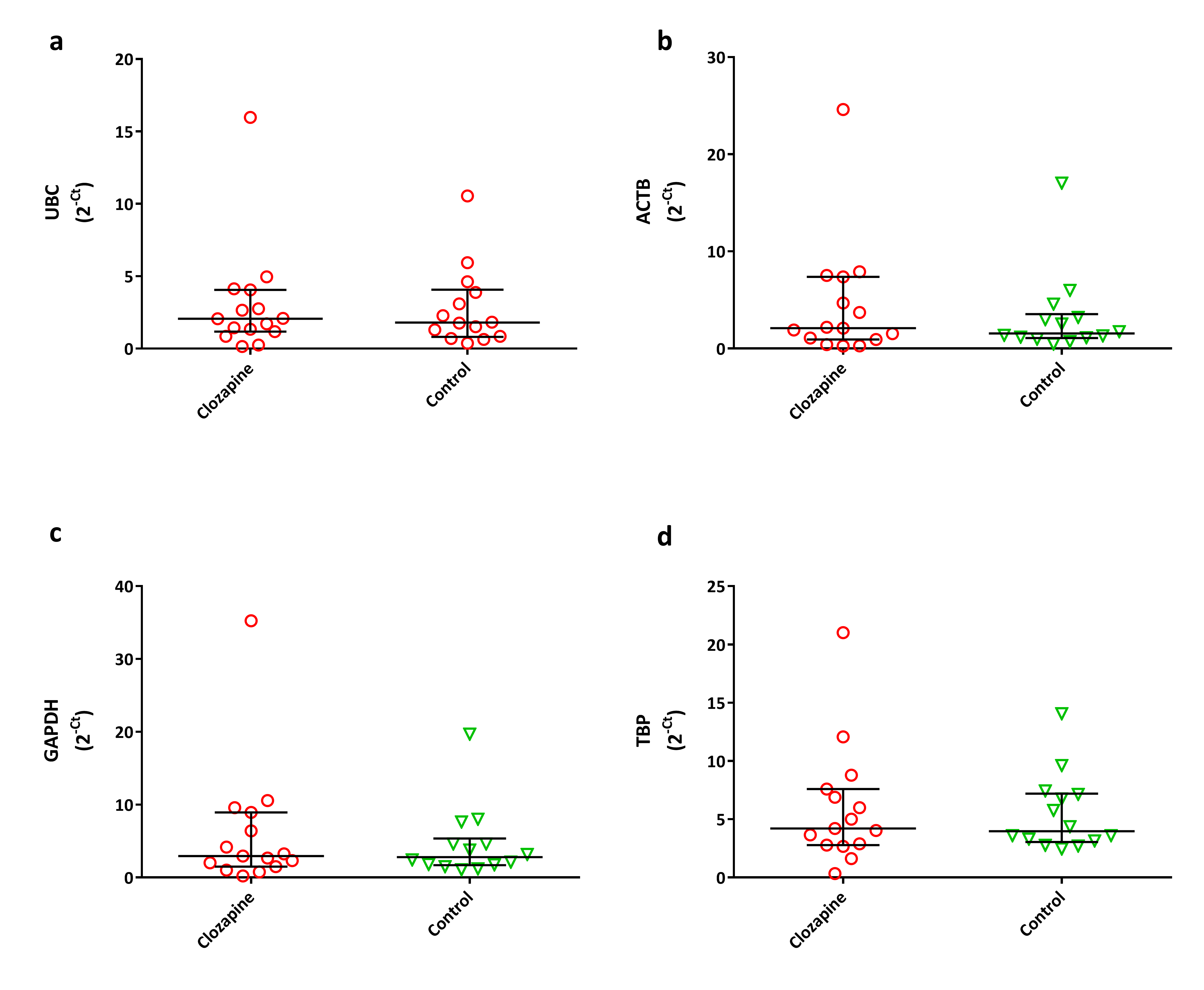


**Supplementary Figure S4:** Expression of reference genes after 7 days clozapine exposure. Wilcoxon signed rank test (matched pair, α=0.05, N=15) was used to measure the difference in reference gene expression between clozapine exposed and control cells; (**a**) UBC (W=-0.722, P=0.47), (**b**) ACTB (W=-0.722, P=0.470), (**c**) GAPDH (W=-1.287, P=0.198), (**d**) TBP (W=-0.909, P=0.363). Error bars represent median ± interquartile range.


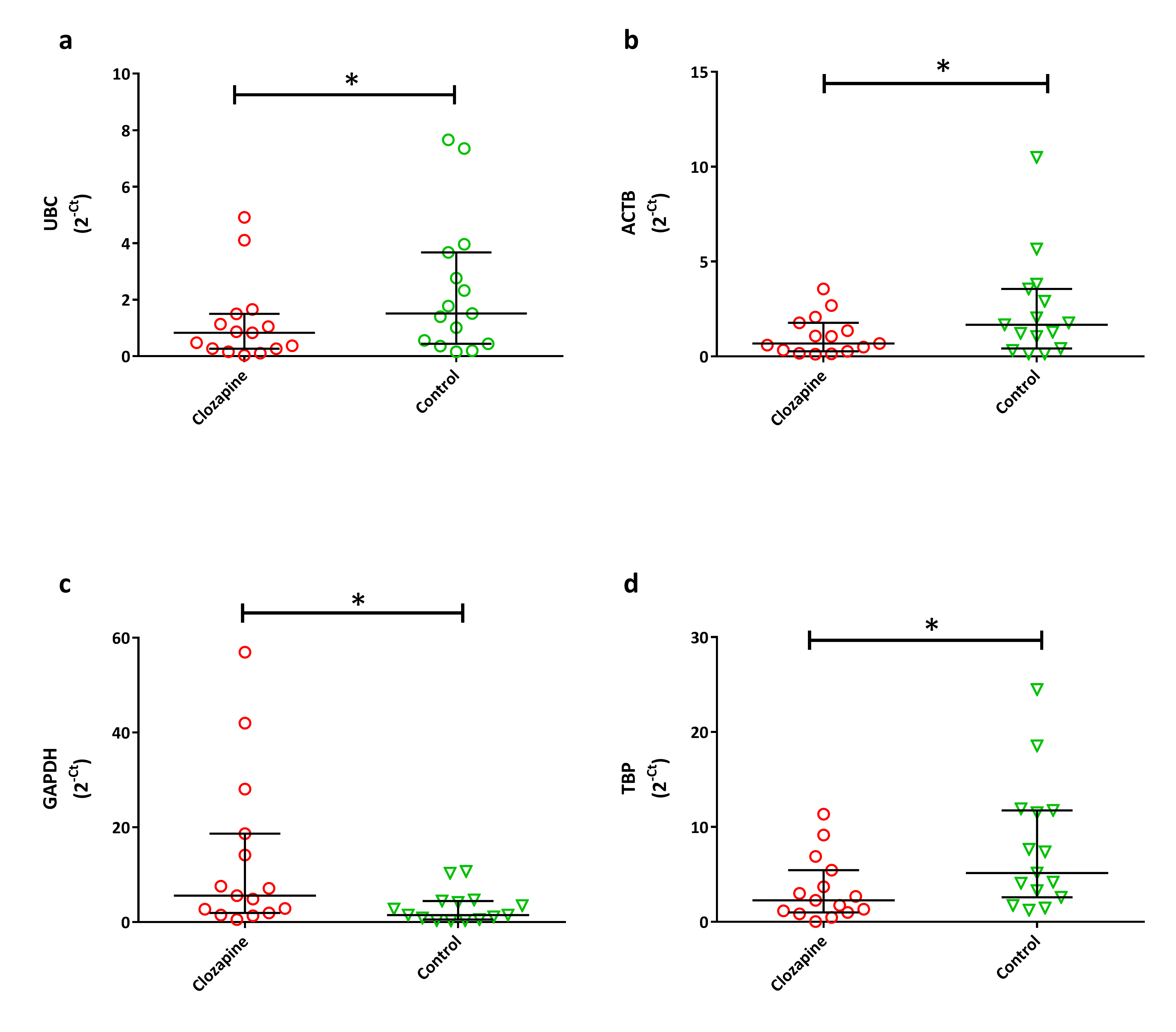


**Supplementary Figure S5:** Expression of reference genes after 24 hour clozapine exposure ; Wilcoxon signed rank test (matched pair, α=0.05, N=15) was used to measure the difference in reference gene expression between clozapine exposed and control cells; (**a**) UBC (W=3.408, P=0.001), (**b**) ACTB (W=3.408, P=0.001), (**c**) GAPDH (W=3.408, P=0.001), (**d**) TBP (W=3.408, P=0.001). Error bars represent median ± interquartile range. *P=0.001.


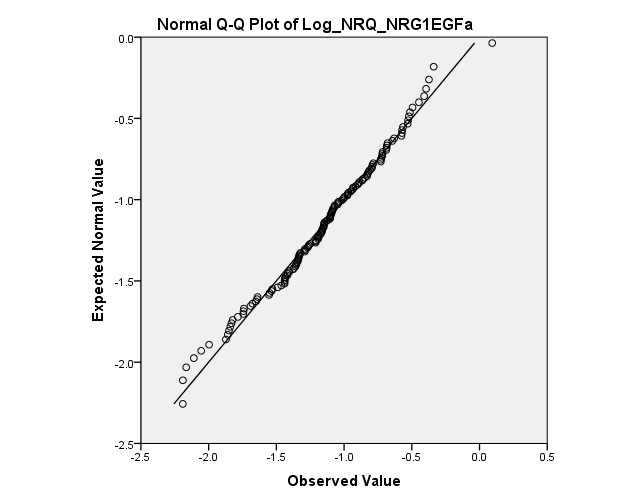

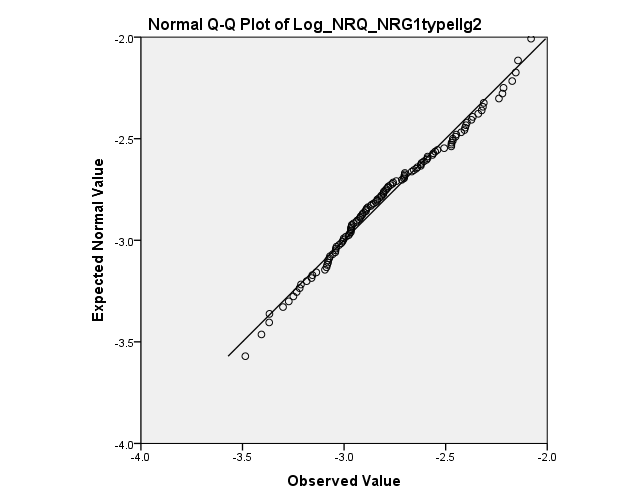


**b**

**d**

**c**

**a**


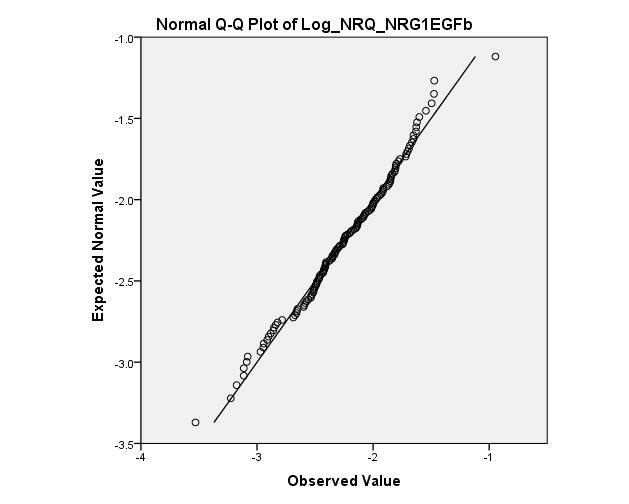

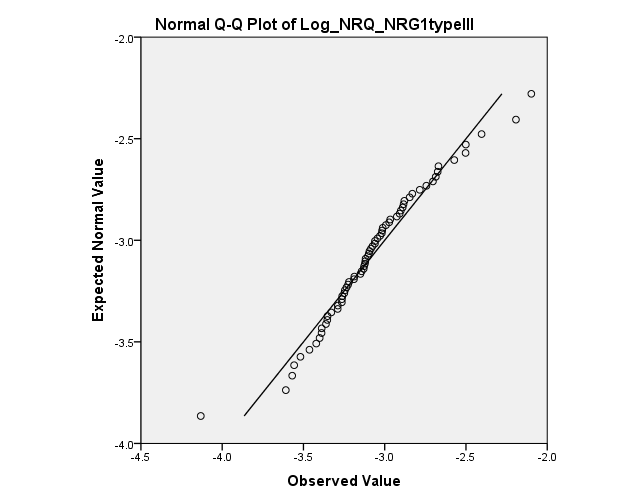


**e**


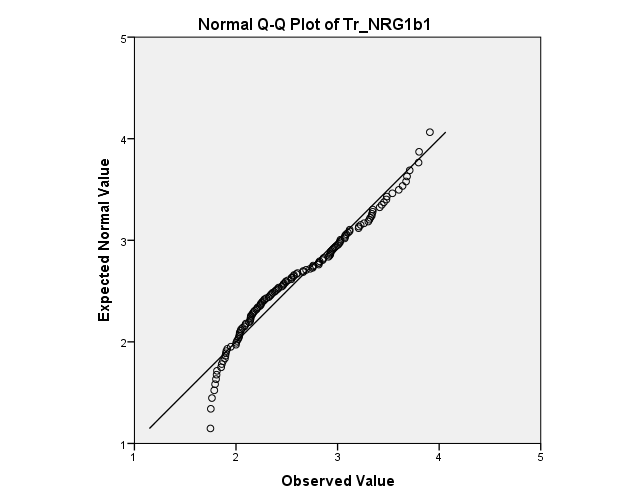


**Supplementary Figure S6**: Quantile-quantile plots of Log10 transformed NRQ values for (a) NRG1EGFα (SW=0.989, df=145, P=0.325), (b) NRG1 typeI_(Ig2)_ (SW=0.986, df=120, P=0.23), (c) NRG1EGFβ (SW=0.99, df=141, P=0.401), (d) NRG1 typeIII (SW=0.968, df=68, P=0.076) and (e) NRG1-β1 (SW=0.957, df=137, P=0.000248). SW=Shapiro-Wilk test.

**
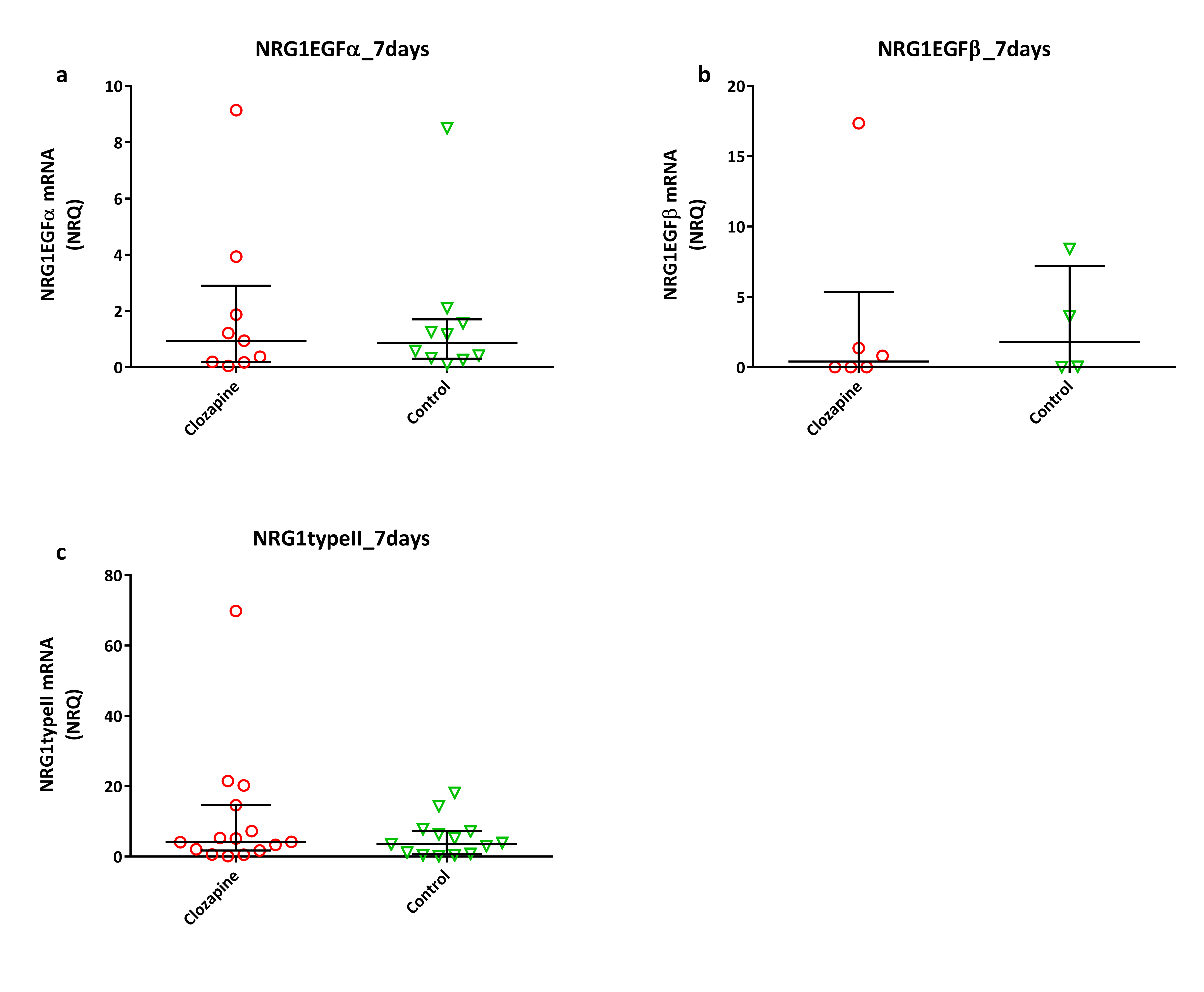
**

**Supplementary Figure S7:** Expression of detectable NRG1 isoforms after 7 days clozapine exposure. Wilcoxon signed rank test (matched pair) was used to measure the difference in the normalized quantity of NRG1 isoforms between clozapine exposed and control cells; (**a**) NRG1EGFα (W=-0.105, P=0.917), (**b**) NRG1EGFβ (W=-0.365, P=0.715), (**c**) NRG1typeII (W=-1.538, P=0.124). Error bars represent median ± interquartile range.


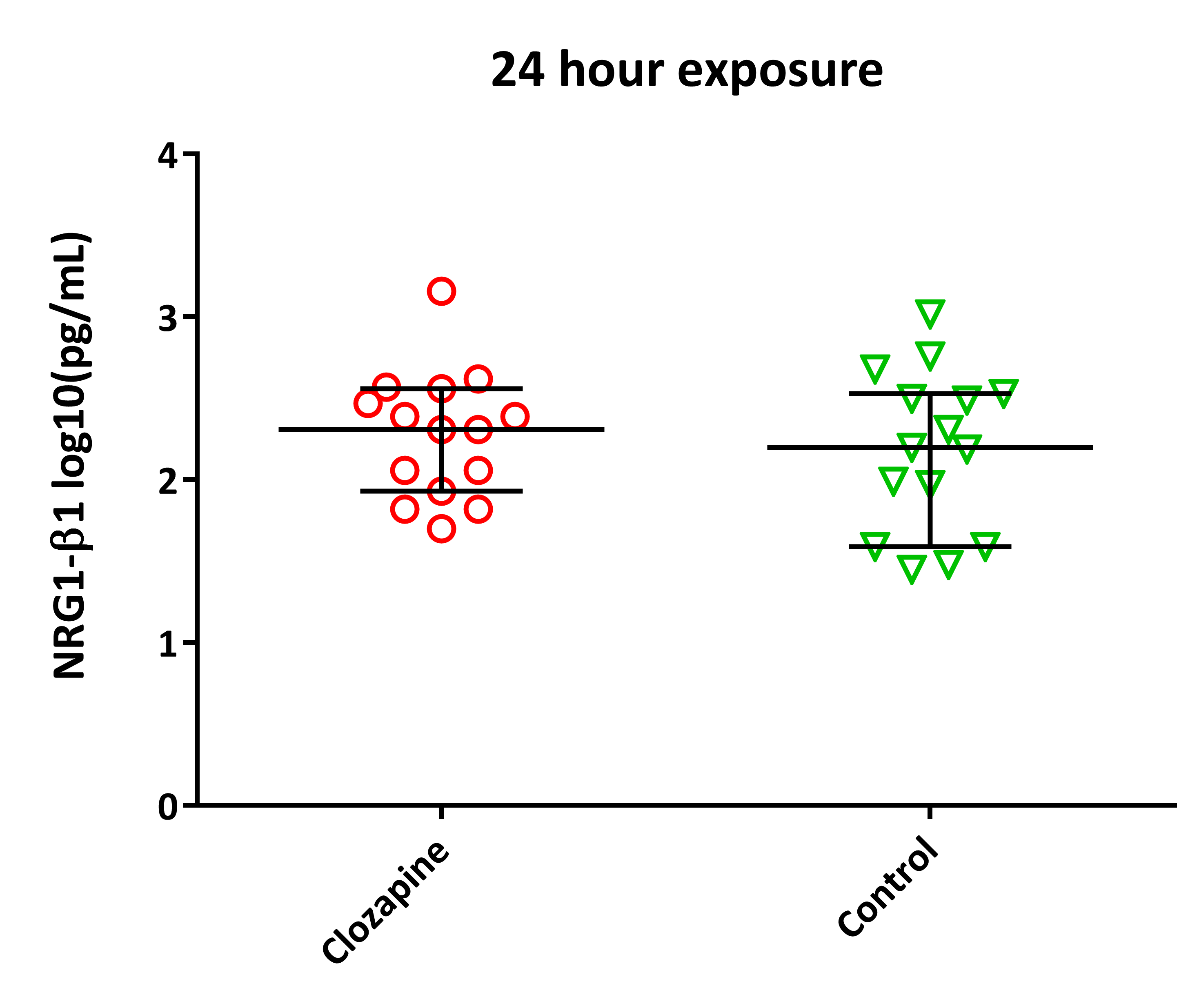


**Supplementary Figure S8:** Expression of NRG1-β1 after 24-hour exposure. Wilcoxon signed rank test (matched pair) was used to measure the difference in NRG1-β1 protein between clozapine exposed and control (W=-1.306, P=0.191). Error bars represent median ± interquartile range.

**
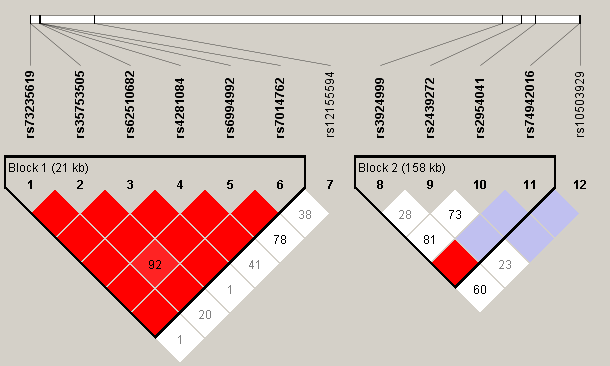

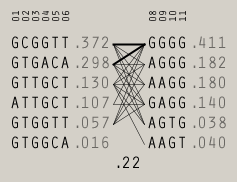
**

**Supplementary Figure S9:** Linkage disequilibrium (D’) between SNPs in the *NRG1* gene. The first block represents strong LD (D’=1.0, r^2^=0.055) between four HapICE SNPs with two other SNPs (rs4281084 and rs7041762) in the 5’ region. The second block represents the LD (D’=1.0, r^2^=0.052) between four SNPs in the 3’ region of the NRG1 gene. The most frequent haplotype in the first block contains each of the risk alleles from the four HapICE SNPs. The most frequent haplotype in the second block contains none of the risk alleles for the corresponding SNPs.

|  | | **Ancestry Informative markers** | | | | | |
| --- | --- | --- | --- | --- | --- | --- | --- |
|  |  | **CEU** |  | **CHB** |  | **YRI** |  |
| **NRG1 SNPs** | **Call rate, %** | **dbSNP** | **Call rate, %** | **dbSNP** | **Call rate, %** | **dbSNP** | **Call rate, %** |
| rs73235619 | 100 | rs1402851 | 99 | rs10488619 | 100 | rs1368928 | 100 |
| rs35753505 | 99 | rs16877243 | 100 | rs11098964 | 100 | rs1446959 | 100 |
| rs62510682 | 100 | rs1698042 | 100 | rs11184898 | 100 | rs1494962 | 100 |
| rs4281084 | 100 | rs2930125 | 100 | rs11203006 | 100 | rs1563382 | 100 |
| rs6994992 | 99 | rs2934193 | 100 | rs1347201 | 100 | rs2388511 | 100 |
| rs7014762 | 100 | rs3912537 | 100 | rs1488299 | 100 | rs4241398 | 99 |
| rs113317778 | Fail | rs4721415 | 100 | rs1519260 | 100 | rs7158302 | 100 |
| rs12155594 | 99 | rs6510332 | 100 | rs1538956 | 96 | rs10933921 | 100 |
| rs2954041 | 100 | rs1001484 | 100 | rs315280 | 99 | rs1444893 | 99 |
| rs6150532 | Fail | rs6552216 | 100 | rs36110 | 100 | rs1716167 | 100 |
| rs3924999 | 100 | rs1002587 | 100 | rs5753625 | 100 | rs1811510 | 100 |
| rs2439272 | 100 | rs10879311 | 100 | rs6595142 | 100 | rs1823778 | 100 |
| rs74942016 | 100 | rs1227647 | 100 | rs12595448 | 100 | rs1827950 | 100 |
| rs10503929 | 100 | rs12678324 | 100 | rs12644851 | 100 | rs1894450 | 100 |
|  |  | rs2759281 | 100 | rs2416504 | 100 | rs2220128 | 100 |
|  |  | rs326626 | 100 | rs2927385 | 99 | rs2416791 | 96 |
|  |  | rs6141319 | 100 | rs4240793 | 100 | rs2948905 | 100 |
|  |  | rs679832 | 100 | rs4265409 | 100 | rs4737761 | 98 |
|  |  | rs10420077 | 100 | rs590614 | 100 | rs6785846 | 100 |
|  |  | rs2102727 | 100 | rs4841401 | 100 | rs692713 | 100 |

**Supplementary Table S1:** NRG1 SNPs with ancestry informative markers and their corresponding call rates. CEU, Northern and Western European; CHB, Han Chinese, Beijing; YRI, Yoruba in Ibadan, Nigeria

| **Gene** | **Probe name** | **Isoforms detected** | **Inventoried assay** | **Forward** | **Reverse** | **Probe**  **(FAM-MGB)** |
| --- | --- | --- | --- | --- | --- | --- |
| NRG1 | Type I_(Ig2)_ | Ig2 and s1 domains (excluding GGF-2, HRG-β1d, HRG-β3b, HRG-γ3, SMDF) | - | GCCAATATCACCATCGTGGAA | CCTTCAGTTGAGGCTGGCATA | CAAACGAGATCATCACTG |
| NRG1 | Type II | GGF-2, HRGβ-1d, HRG-β3b, HRG-γ3 | - | GAATCAAACGCTACATCTACATCCA | CCTTCTCCGCACATTTTACAAGA | CACTGGGACAAGCC |
| NRG1 | Type IV | HRG-β1b, HRG-β1c & HRG-β1d | - | GCTCCGGCAGCAGCAT | GAACCTGCAGCCGATTCCT | ACCACAGCCTTGCCT |
| NRG1 | EGFα | HRG-α, ndf43 and ndf43c | Hs01103794_m1 | - | - | - |
| NRG1 | EGFβ | SMDF, GGF, GGF2, ndf43a, ndf43b and all HRG-β isoforms | Hs00247624_m1 | - | - | - |
| NRG1 | Type III | SMDF | Hs01103792_m1 | - | - | - |
| NRG1 | pan-NRG1 | all isoforms (excluding SMDF, ndf43c) | Hs00247620_m1 | - | - | - |
| NRG1 | Type I | HRG-β3b and HRG-γ3 (excluding HRG-β1b, HRG-β1c) | Hs01108479_m1 | - | - | - |
| UBC | - | ubiquitin C | Hs00824723_m1 | - | - | - |
| GAPDH | - | glyceraldehyde-3-phosphate dehydrogenase | Hs99999905_m1 | - | - | - |
| ACTB | - | beta-actin | Hs99999903_m1 | - | - | - |
| TBP | - | TATA box binding protein | Hs00427620_m1 | - | - | - |

**Supplementary Table S2**: TaqMan probes and primer sequence for quantification and normalization of different NRG1 isoform expression. Custom designed probe and primer combinations were used to target specific NRG1 isoforms previously investigated ^2^. For all the other isoforms, inventoried assays were used (Applied Biosystems, Foster City, CA, USA). The geometric mean of ACTB and UBC were used for transcript normalization.

| **NRG1 isoforms/ protein** | **Alcohol Use**  **N; mean (se)** | **No Alcohol Use**  **N; mean (se)** | **P value** |
| --- | --- | --- | --- |
| NRG1EGFα | 113; 1.12 (0.13) | 13; 1.25 (0.25) | 0.75 |
| NRG1 typeI_(Ig2)_ | 93; 2.10 (0.17) | 11; 1.61 (0.24) | 0.39 |
| NRG1EGFβ | 111; 9.17 (1.18) | 12; 10.74 (2.02) | 0.67 |
| NRG1 typeIII | 52; 12.48 (1.98) | 08; 8.01 (1.33) | 0.39 |
| NRG1-β1 log10(pg/mL) | 108; 2.65 (0.05) | 12; 2.63 (0.20) | 0.89 |

**Supplementary Table S3:** Normalized relative quantities (NRQ) of detectable *NRG1* mRNA isoforms and NRG1-β1 by alcohol use (past 3-months).

|  | **Non-detects, n (%)** | | |  |
| --- | --- | --- | --- | --- |
| **Gene** | **Total (128)** | **Schizophrenia (71)** | **Controls (57)** | **P value** |
| NRG1 EGFα | 2 (1.6) | 1 (1.4) | 1 (1) | 0.87 |
| NRG1 EGFβ | 5 (3.9) | 3 (4.2) | 2 (3.5) | 0.83 |
| NRG1 type I_(Ig2)_ | 24 (18.8) | 11 (15.5) | 13 (22.8) | 0.29 |
| NRG1 typeIII | 68 (53.1) | 33 (46.5) | 35 (61.4) | 0.09 |
| Pan NRG1 | 100 (78.1) | 51 (71.8) | 49 (86) | 0.06 |
| NRG1 type I | 109 (85.2) | 57 (80.3) | 52 (91.2) | 0.08 |
| NRG1 type II | 88 (68.8) | 39 (54.9) | 49 (86) | 0.00016* |
| NRG1 type IV | 128 (100) | 71 (100) | 57 (100) | 0 |

**Supplementary Table S4:** Number of non-detects n (%) for all *NRG1* isoforms assessed. Z-test was performed to find differences between schizophrenia and controls. *P<0.05

| **Variables** | **NRG1 EGFα (n=70)** | **NRG1 typeI_(Ig2)_ (n=60)** | **NRG1 EGFβ (n=68)** | **NRG1**  **typeIII**  **(n=38)** | **NRG1-β1 (n=70)** |
| --- | --- | --- | --- | --- | --- |
| Clozapine plasma level (µg/L) | 0.08  (0.513) | 0.08  (0.538) | 0.19  (0.134) | -0.02  (0.929) | -0.02  (0.851) |
| Chlorpromazine equivalent antipsychotic exposure | -0.03  (0.806) | 0.08  (0.570) | -0.06  (0.647) | -0.05  (0.768) | -0.01  (0.925) |
| Age of onset, years | -0.37  (**0.002, 0.02**) | -0.05 (0.708) | -0.38  (**0.001**, **0.02**) | 0.19  (0.250) | 0.18 (0.142) |
| Duration of illness, years | 0.01  (0.964) | -0.22  (0.094) | 0.05  (0.696) | -0.36  (**0.027**, 0.167) | -0.11 (0.375) |

**Supplementary Table S5:** Pearson’s correlation (raw P-value, Benjamini-Hochberg adjusted P- value) between different NRG1 isoforms and NRG1-β1 with clozapine plasma level and chlorpromazine equivalent antipsychotic exposure (excluding clozapine).

| **Transcript** | **Positive**  **score** | **Negative score** | **Disorganized score** | **Excitement score** | **Depression score** | **Total score** |
| --- | --- | --- | --- | --- | --- | --- |
| NRG1EGFα (n=70) | -0.02  (0.903) | 0.020 (0.867) | -0.012  (0.921) | 0.160  (0.185) | 0.241  (**0.045**, 0.270) | -0.014 (0.908) |
| NRG1typeI_(Ig2)_ (n=60) | -0.169 (0.197) | 0.112 (0.393) | -0.067  (0.612) | -0.014  (0.918) | -0.057  (0.664) | -0.015 (0.909) |
| NRG1EGFβ (n=68) | -0.057 (0.643) | 0.085 (0.490) | -0.043  (0.726) | 0.127  (0.303) | -0.227  (0.063) | -0019 (0.881) |
| NRG1typeIII (n=38) | -0.377  (**0.020**, 0.120) | 0.257 (0.119) | -0.129  (0.441) | -0.130  (0.436) | -0.182  (0.275) | -0.133 (0.427) |
| NRG1-β1 (n=70) | -0.01  (0.942) | 0.11 (0.348) | -0.15  (0.213) | -0.10  (0.399) | 0.19  (0.111) | 0.05 (0.689) |

**Supplementary Table S6:** Pearson’s correlation (raw P-value, Benjamini-Hochberg adjusted P- value) between NRG1 isoforms and NRG1-β1 protein level with PANSS positive, negative, disorganized, excited, depression score and total score.

| **NRG1 isoforms/ protein** | **Remission**  **N; mean (se)** | **Non-remission**  **N; mean (se)** | **Raw P value** | **BH P value*** |
| --- | --- | --- | --- | --- |
| NRG1EGFα | 31; 1.35 (0.23) | 39; 1.27 (0.13) | 0.525 |  |
| NRG1 typeI_(Ig2)_ | 26; 2.70 (0.37) | 34; 1.70 (0.23) | 0.303 |  |
| NRG1EGFβ | 29; 12.4 (1.89) | 39; 9.9 (1.01) | 0.511 |  |
| NRG1 typeIII | 13; 18.7 (5.56) | 25; 8.82 (1.47) | **0.013** | 0.065 |
| NRG1-β1 log10(pg/mL) | 31; 2.57(0.11) | 39; 2.39(0.10) | 0.239 |  |

**Supplementary Table S7:** Normalized relative quantities (NRQ) of detectable *NRG1* mRNA isoforms and NRG1-β1 by positive symptom remission status. Positive symptom remission was defined as a score of ≤3 on four PANSS items (delusions, hallucinations, grandiosity and unusual thought content) ^6^. *Benjamini-Hochberg adjusted P-value

| **SNP** | **NRG1EGFα (n=126)** | **NRG1typeI_(Ig2)_**  **(n=104)** | **NRG1EGFβ**  **(n=123)** | **NRG1TypeIII**  **(n=60)** | **NRG1-β1**  **(n=120)** |
| --- | --- | --- | --- | --- | --- |
| rs73235619 | 5.278  (0.207) | 0.006  (0.964) | 1.661  (0.523) | 3.633  (0.295) | 0.191  (0.867) |
| rs35753505 | 1.614  (0.522) | 0.212  (0.861) | 0.271  (0.861) | 0.431  (0.849) | 4.855  (0.207) |
| rs62510682 | 2.814  (0.330) | 0.240  (0.861) | 2.470  (0.377) | 3.488  (0.299) | 5.198  (0.207) |
| rs4281084 | 0.330  (0.861) | 0.452  (0.849) | 1.412  (0.529) | 0.033  (0.957) | SZ: 0.626 (0.764)  Con: 6.475 (0.207) |
| rs6994992 | 1.507  (0.522) | 0.876  (0.686) | 0.002  (0.967) | 2.800  (0.331) | 8.757 (P=0.152) |
| rs7014762 | 0.004  (0.964) | 1.205  (0.579) | 0.789  (0.696) | 0.080  (0.910) | SZ: 0.814 (0.697)  Con: 7.749(0.203) |
| rs12155594 | SZ: 0.213 (0.861)  Con: 4.557(0.211) | 0.706  (0.729) | 0.024  (0.963) | 0.004  (0.963) | 0.067  (0.916) |
| rs3924999 | 8.641  (0.152) | 0.008  (0.964) | 2.801  (0.330) | 0.110  (0.899) | 0.238  (0.861) |
| rs2439272 | 0.238  (0.861) | 0.179  (0.867) | 1.082  (0.616) | 0.338  (0.861) | 0.083  (0.910) |
| rs2954041 | 3.029  (0.331) | 0.128  (0.898) | 2.854  (0.330) | 0.016  (0.964) | SZ: 3.368 (0.299)  Con: 4.487(0.212) |
| rs74942016 | 0.039  (0.956) | 0.257  (0.861) | 1.476  (0.523) | 2.210  (0.403) | 2.173  (0.403) |
| rs10503929 | SZ: 4.986 (0.207)  Con: 4.610 (0.211) | 0.106  (0.899) | SZ: 5.876 (0.207)  Con: 2.414 (0.386) | 0.229  (0.861) | 1.536  (0.523) |
| Hap GCGT | 1.210  (0.579) | 0.484  (0.843) | 0.129  (0.898) | 0.947  (0.67) | 5.142  (0.207) |
| Hap ATTC | 5.598  (0.207) | 0.004  (0.964) | 1.813  (0.491) | 3.633  (0.295) | 0.236  (0.861) |

**Supplementary Table S8:** Expression quantitative trait loci (eQTL) analysis of putative NRG1 SNPs with NRG1 isoforms and NRG1-β1 protein expression. F value (Benjamini-Hochberg adjusted P-value) is shown for each SNP. In cases where a significant SNP x group interaction was detected, F values (p-values) are presented for schizophrenia and control groups separately.

**References:**

1. Chen ML, Tsai TC, Lin YY, Tsai YM, Wang LK, Lee MC*, et al*. Antipsychotic drugs suppress the AKT/NF-kappaB pathway and regulate the differentiation of T-cell subsets. *Immunol Lett* 2011; **140**(1-2)**:** 81-91.

2. Weickert CS, Tiwari Y, Schofield PR, Mowry BJ, Fullerton JM. Schizophrenia-associated HapICE haplotype is associated with increased NRG1 type III expression and high nucleotide diversity. *Translational psychiatry* 2012; **2:** e104.

3. Law AJ, Lipska BK, Weickert CS, Hyde TM, Straub RE, Hashimoto R*, et al*. Neuregulin 1 transcripts are differentially expressed in schizophrenia and regulated by 5' SNPs associated with the disease. *Proc Natl Acad Sci U S A* 2006; **103**(17)**:** 6747-6752.

4. Hashimoto R, Straub RE, Weickert CS, Hyde TM, Kleinman JE, Weinberger DR. Expression analysis of neuregulin-1 in the dorsolateral prefrontal cortex in schizophrenia. *Molecular psychiatry* 2004; **9**(3)**:** 299-307.

5. Weickert CS, Sheedy D, Rothmond DA, Dedova I, Fung S, Garrick T*, et al*. Selection of reference gene expression in a schizophrenia brain cohort. *Aust N Z J Psychiatry* 2010; **44**(1)**:** 59-70.

6. Wallwork RS, Fortgang R, Hashimoto R, Weinberger DR, Dickinson D. Searching for a consensus five-factor model of the Positive and Negative Syndrome Scale for schizophrenia. *Schizophr Res* 2012; **137**(1-3)**:** 246-250.
